# Mechanisms of thyrotropin receptor–mediated phenotype variability deciphered by gene mutations and M453T-knockin model

**DOI:** 10.1172/jci.insight.167092

**Published:** 2024-01-09

**Authors:** Kristiina Makkonen, Meeri Jännäri, Luís Crisóstomo, Matilda Kuusi, Konrad Patyra, Vladyslav Melnyk, Veli Linnossuo, Johanna Ojala, Rowmika Ravi, Christoffer Löf, Juho-Antti Mäkelä, Päivi Miettinen, Saila Laakso, Marja Ojaniemi, Jarmo Jääskeläinen, Markku Laakso, Filip Bossowski, Beata Sawicka, Karolina Stożek, Artur Bossowski, Gunnar Kleinau, Patrick Scheerer, FinnGen FinnGen, Mary Pat Reeve, Jukka Kero

**Affiliations:** 1 Department of Clinical Sciences, Faculty of Medicine, and; 2 Integrative Physiology and Pharmacology, Institute of Biomedicine, University of Turku, Turku, Finland.; 3 New Children’s Hospital, Helsinki University Hospital, Helsinki, Finland.; 4 Department of Pediatrics and Adolescence, PEDEGO Research Unit and Medical Research Center, University and University Hospital of Oulu, Oulu, Finland.; 5 Department of Pediatrics, Kuopio University Hospital, Kuopio, Finland.; 6 Institute of Clinical Medicine, Internal Medicine, University of Eastern Finland, Kuopio, Finland.; 7 Department of Pediatrics, Endocrinology, Diabetes with a Cardiology Unit, Medical University in Białystok, Bialystok, Poland.; 8 Charité - Universitätsmedizin Berlin, Corporate Member of Freie Universität Berlin, and; 9 Humboldt - Universität zu Berlin, Institute of Medical Physics, Biophysics, Group Structural Biology of Cellular Signaling, Berlin, Germany.; 10 Institute for Molecular Medicine Finland, HiLIFE, University of Helsinki, Helsinki, Finland.; 11 FinnGen is detailed in Supplemental Acknowledgments.; 12 Department of Pediatrics and Adolescent Medicine, Turku University Hospital, Turku, Finland.

**Keywords:** Endocrinology, Genetics, G protein&ndash;coupled receptors, Genetic diseases, Thyroid disease

## Abstract

The clinical spectrum of thyrotropin receptor–mediated (TSHR-mediated) diseases varies from loss-of-function mutations causing congenital hypothyroidism to constitutively active mutations (CAMs) leading to nonautoimmune hyperthyroidism (NAH). Variation at the TSHR locus has also been associated with altered lipid and bone metabolism and autoimmune thyroid diseases. However, the extrathyroidal roles of TSHR and the mechanisms underlying phenotypic variability among TSHR-mediated diseases remain unclear. Here we identified and characterized TSHR variants and factors involved in phenotypic variability in different patient cohorts, the FinnGen database, and a mouse model. TSHR CAMs were found in all 16 patients with NAH, with 1 CAM in an unexpected location in the extracellular leucine-rich repeat domain (p.S237N) and another in the transmembrane domain (p.I640V) in 2 families with distinct hyperthyroid phenotypes. In addition, screening of the FinnGen database revealed rare functional variants as well as distinct common noncoding TSHR SNPs significantly associated with thyroid phenotypes, but there was no other significant association between TSHR variants and more than 2,000 nonthyroid disease endpoints. Finally, our TSHR M453T–knockin model revealed that the phenotype was dependent on the mutation’s signaling properties and was ameliorated by increased iodine intake. In summary, our data show that TSHR-mediated disease risk can be modified by variants at the TSHR locus both inside and outside the coding region as well as by altered TSHR-signaling and dietary iodine, supporting the need for personalized treatment strategies.

## Abstract 2

<p>The clinical spectrum of thyrotropin receptor-mediated diseases varies widely&nbsp;and disease risk can be modified by several factors including signaling properties of thyrotropin receptor mutations and dietary iodine.</p>

## Graphical abstract

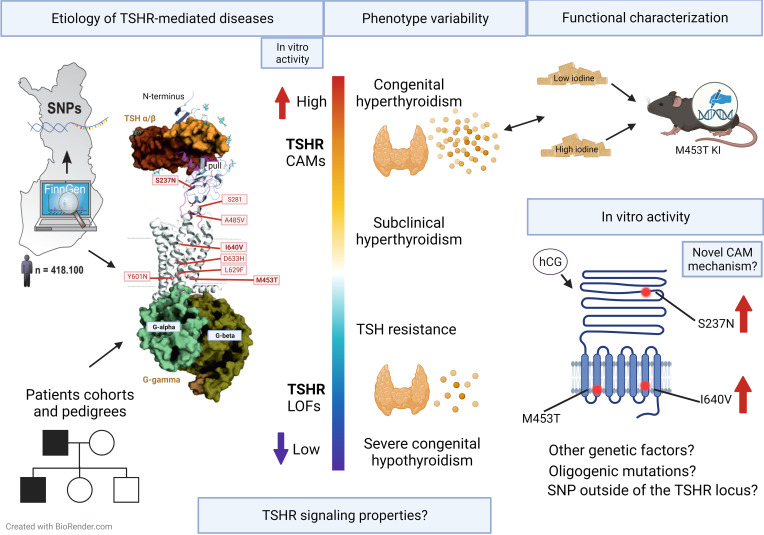

## Introduction

Thyroid-stimulating hormone (TSH) is the primary regulator of thyroid growth and function (1). Lack of TSH or its action due to mutations inactivating the TSH receptor (TSHR) impairs thyroid function, leading to hypothyroidism. In contrast, pathologically elevated serum TSH levels, TSHR-stimulating antibodies, or constitutively active TSHR mutations stimulate thyroid hormone production and thyroid growth, resulting in hyperthyroidism and goiter (2). TSH regulates thyroid function via TSHR, a G protein–coupled receptor (GPCR), which preferentially couples to the α-subunit of the stimulatory guanine-nucleotide-binding protein (Gα_s_) that, in turn, activates adenylate cyclase and increases intracellular cyclic AMP (cAMP) (1, 3). However, higher TSH concentrations can also activate Gα_q/11_-mediated signaling, resulting in the activation of phospholipase C and an increase in intracellular calcium levels (4). This signaling pathway is thought to be involved in TSH resistance phenotypes in some patients (5, 6) and in murine goiter development (7). Nevertheless, unlike some other GPCRs (8) the physiological effect of the different TSHR-mediated G protein signaling remains unclear.

Mutations in TSHR are associated with a wide range of diseases (2). They can be either somatic or germline, present as gain- or loss-of-function mutations, and lead to nonautoimmune hyperthyroidism (NAH) or variable degrees of hypothyroidism, respectively (9–11). Gain of function due to a constitutively active mutation (CAM) in TSHR is the most common cause of NAH (11), which includes familial NAH (FNAH) and sporadic congenital NAH (SNAH). TSHR CAMs are also found in up to 80% of toxic thyroid nodules and toxic multinodular goiters (12). To date, over 40 families and 23 sporadic case reports of TSHR CAMs have been published (10). Typically, these TSHR CAMs lead to ligand-independent TSHR activity, an increase in thyroid hormone synthesis, and hyperthyroidism, which is usually persistent and requires ablative therapy in order for patients to avoid relapse (11). Therefore, it is recommended that all patients with FNAH be evaluated for TSHR-activating mutations.

In addition to the principal role of TSHR in thyroid, extrathyroidal expression of TSHR has been observed in several tissues. TSHR is reported to be an important regulator of Graves’ orbitopathy and adipocyte differentiation (13–15), to affect thermogenesis (16, 17) and osteoblast differentiation (18), and to negatively regulate osteoclastogenesis (19, 20). However, there is still a knowledge gap concerning these possible direct extrathyroidal effects of TSHR.

Furthermore, our previous findings confirm the primary roles of Gα_s_- and Gα_q/11_-mediated signaling in the thyrocytes, as we have used different mouse models to investigate the role of G protein signaling in the pathogenesis of TSHR-mediated disease. In these models, we tested the effect of thyrocyte-specific G protein deficiencies on thyroid function and growth (21–23), and we observed that only thyrocyte-specific Gα_s_-deficient mice developed acute hypothyroidism (22). While mice lacking Gα_q/11_-mediated signaling in the thyroid had impaired thyroid growth but developed subclinical to overt hypothyroidism only at older ages (7), the conditional Gα_12/13_-deficient mice had no obvious phenotypes (21). Surprisingly, strong constitutive activity of both Gα_s_ and Gα_q/11_, detected in our recently developed NAH mouse model (23), carrying a patient-derived TSHR D633H mutation (24) led to only relatively mild and transient hyperthyroidism but advanced to papillary thyroid carcinoma (PTC). Therefore, the data from this model suggest that NAH might be a dynamic condition dependent on individual receptor mutant properties.

In humans, the role of distinct TSHR CAMs in the pathogenesis of hyperthyroidism remains unclear (25–27). Consequently, in this study, we genetically and clinically evaluated patient cohorts and the FinnGen project data to identify TSHR variants and other genetic variations involved in disease variability. We also screened FinnGen to identify other disease associations at TSHR across the full clinical spectrum of disease endpoints and performed functional analysis for some of the identified TSHR variants. We also generated a TSHR M453T–knockin mouse model to elucidate potential factors that may underly the phenotypic variability of TSHR-mediated diseases.

## Results

### Clinical characteristics of FNAH and SNAH, and cases with toxic thyroid nodules.

**Figure 1:**
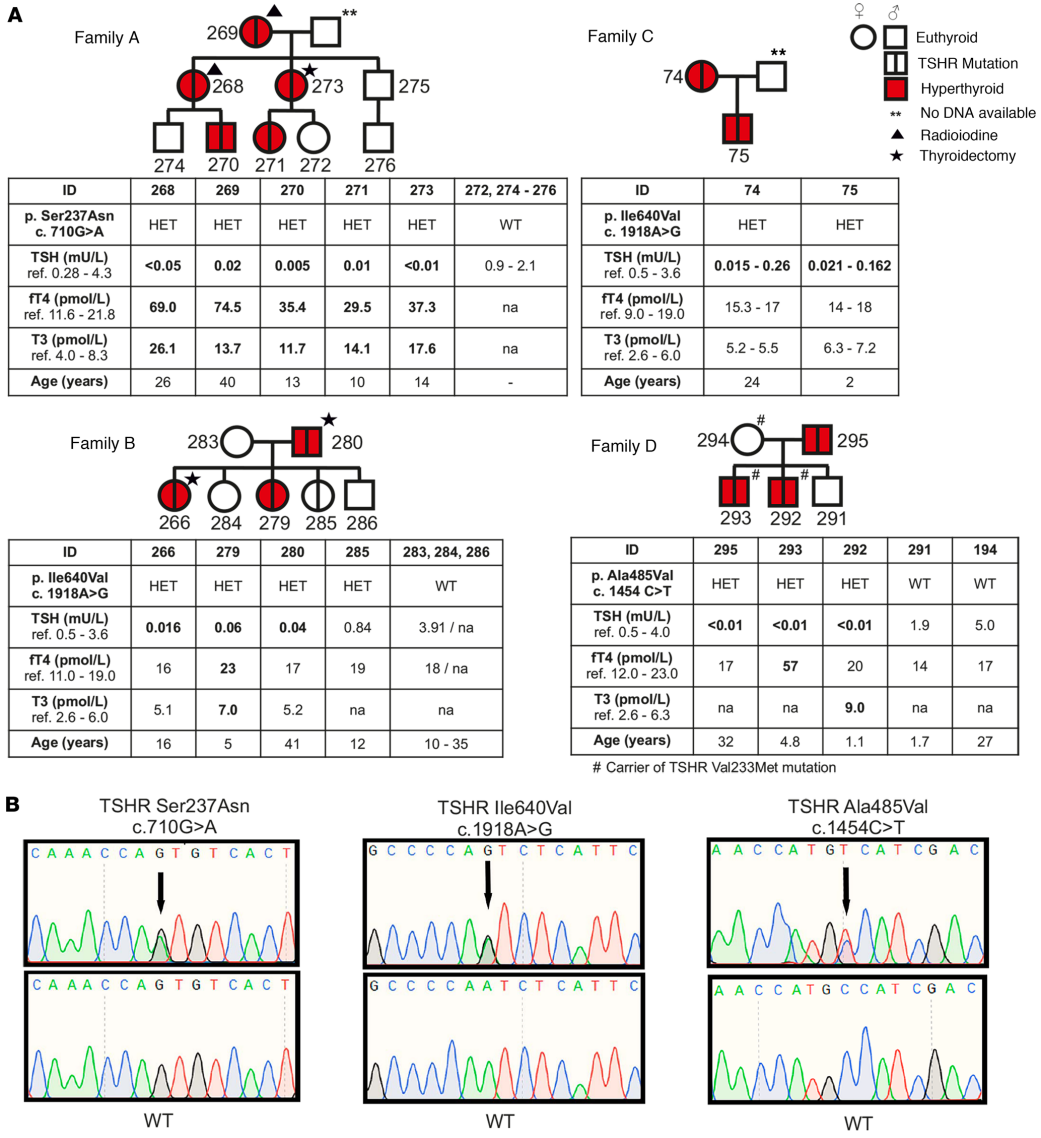
Phenotypic, genotypic, and clinical data of 4 modified pedigrees with familial nonautoimmune hyperthyroidism. (**A**) Illustrates pedigrees of 4 kindreds with overt nonautoimmune hyperthyroidism across 3 generations with an extracellular TSHR Ser237Asn (p.S237N) mutation among all affected cases of Family A. Families B and C are nonconsanguineous Finnish kindreds with a TSHR Ile640Val (p.I640V) mutation in all affected cases presenting overt (Family B) and subclinical (Family C) hyperthyroidism (patient #285 is euthyroid carrier). Family D shows individuals with subclinical to overt hyperthyroidism and a TSHR Ala485Val (p.A485V) mutation in 3 affected family members. (**B**) Representative chromatograms of the identified mutations and their corresponding WT family members. The mutation, zygosity, age at diagnosis, and serum TSH, free thyroxine (fT4) and free triiodothyronine (fT3) hormone levels at the time of diagnosis are listed below the pedigree, except for Family C, for which the ranges of TSH, fT4, and fT3 serum levels during several years of follow-up are shown. ** indicates no DNA available; star indicates radioiodine; and triangle indicates thyroidectomy treatments; # indicates that this person is a carrier of the TSHR Val233Met mutation. Hyperthyroid cases are marked with red color, and mutation carriers are marked with vertical line. In 2 individuals (patients #266 and #279) of Family B, hyperthyroidism was activated during the first trimester of pregnancy. Patients #74, #75, and #295 have subclinical hyperthyroidism and have not required antithyroid treatment during 20 (patient #74), 10 (patient #75), or 2 (patient #295) years of follow-up.

We studied 4 different families with NAH (Figure 1). The clinical characteristics of the pedigrees are described in more detail in the Supplemental Methods (supplemental material available online with this article; https://doi.org/10.1172/jci.insight.167092DS1). Briefly, family A was nonconsanguineous kindred of Polish origin, with overt NAH across 3 generations. The age at presentation of clinical symptoms and diagnosis ranged from 10 to 40 years. All patients had plasma TSH (P-TSH) and free thyroxine (fT4) concentrations below and above the reference range, respectively, and undetectable levels of TSHR antibodies at diagnosis. Three family members (patients #268, #269, and #273) showed persistent hyperthyroidism and underwent thyroidectomy and radioiodine treatments. There was no history of osteoporosis or fractures among the family members, and all other biochemical tests were normal in hyperthyroid cases (Figure 1 and Supplemental Table 1).

Families B and C were Finnish nonconsanguineous kindreds. Every child had normal umbilical TSH values at neonatal screening for congenital hypothyroidism (Supplemental Table 1). All affected individuals were initially diagnosed with subclinical hyperthyroidism and had suppressed serum TSH but had normal fT4 and free triiodothyronine (fT3) levels. Interestingly, 2 individuals (patients #266 and #279) of family B had mostly subclinical hyperthyroidism prior to pregnancy, and it rapidly worsened during the first trimester of pregnancy with an increase in fT4 and fT3 levels and required treatment adjustment (Supplemental Figure 1). The thyroid histology of patients #266 and #280 revealed typical thyroid hyperplasia with variable-sized follicles (Supplemental Figure 2).

In the family C, the index patient (patient #74) underwent thyroid function tests due to essential hypertension at 24 years old. At diagnosis of subclinical hyperthyroidism, her TSH concentration was below detection limit (0.06 mU/L, range: 0.5–3.6 mU/L), but fT4 (17 pmol/L, reference: 9.0–19 pmol/L) and fT3 (5.5 pmol/L, reference: 2.6–6 pmol/L) concentrations were in range (Figure 1). The patient had no hyperthyroid symptoms, and thyroid imaging, bone density, or fT4 levels remained within the normal ranges over 20 years of follow-up. The patient’s son (patient #75) also revealed subclinical NAH with normal thyroid imaging, bone age, linear growth, BMI, head growth, weight gain, and neurological development (Supplemental Figure 3 and Supplemental Table 1).

In the family D, the index case (patient #293) and his brother developed overt symptomatic NAH at the age of 13 months and 3 years, respectively, while the patients’ mother (patient #294) and the other sibling (patient #291) were asymptomatic. At the time of recruitment, his father (patient #295) had low serum TSH but normal fT4 concentration with no hyperthyroid symptoms (Figure 1).

**Figure 2:**
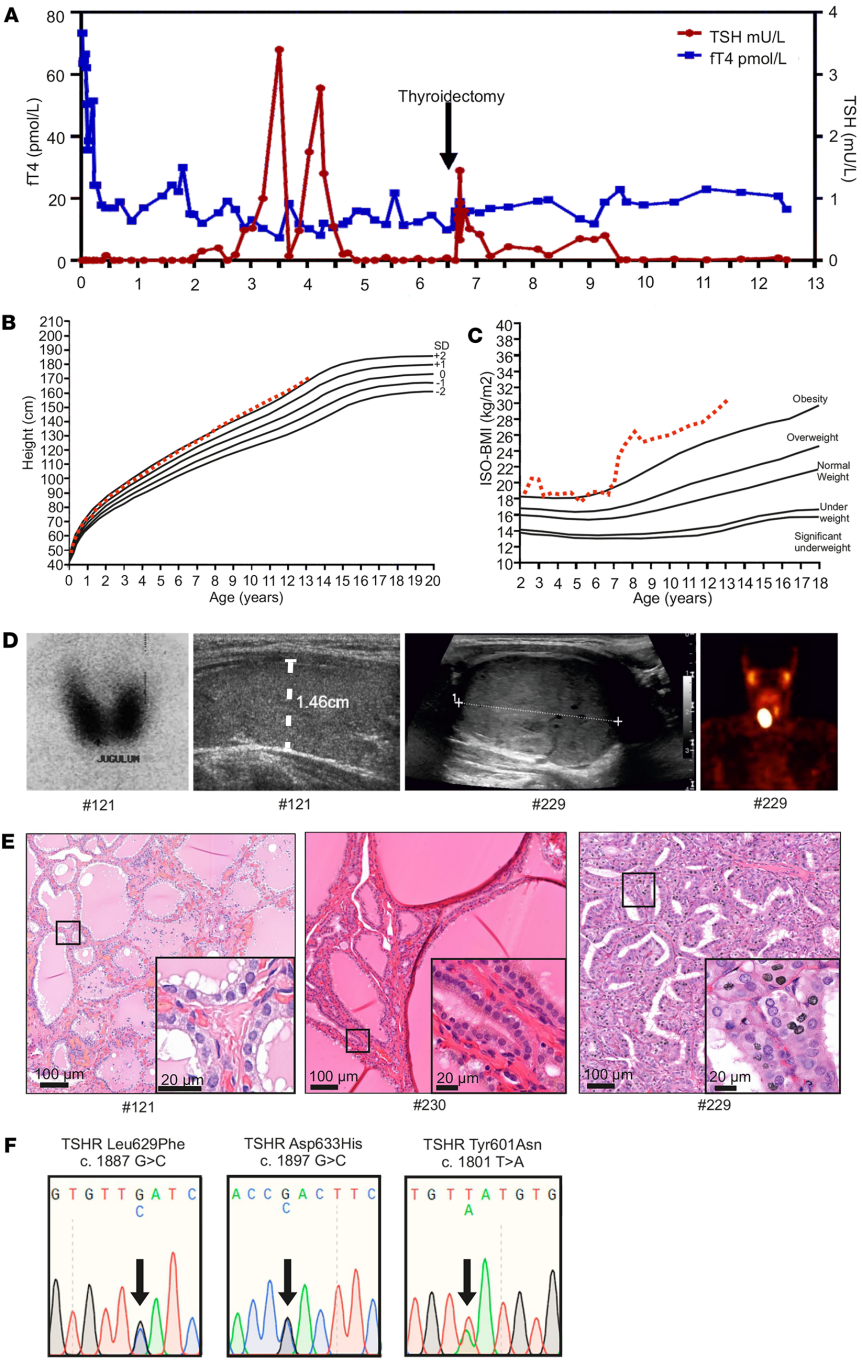
Thyroid function tests, clinical data, imaging results, and genetic data of the patients with de novo germline constitutive active TSHR Leu629Phe mutation and somatic TSHR Asp633His and Tyr601Asn mutations. (**A**) Serum thyrotropin (TSH) and free thyroxine (fT4) concentrations of patient #121 from birth to 12 years of age. (**B** and **C**) Height-BMI and ISO-BMI curves. (**D**) Iodine uptake and thyroid ultrasound image at 2 months of age showing high and homogenous radioactivity accumulation in both thyroid lobes and normal-sized thyroid (diameter indicated with white dotted line) in patient #121. Thyroid ultrasound image and single photon emission computed tomography (SPECT) imaging showing exact boundaries of solid thyroid nodule on the right thyroid lobe and large oval accumulation of ^99m^Tc on the right side of thyroid of patient #229. (**E**) Histological analysis of the H&E-stained thyroid tissue of patients #121, #230, and #229. (**F**) Representative illustration of Sanger sequencing chromatogram of the patients carrying TSHR Leu629Phe (c.1887G>C, p.l629F), Asp633His (c.1897G>C, p.D633H), and Tyr601Asn (c.1801T>A, p.Y601N) mutations. ISO-BMI, age and sex-adjusted body mass index.

In addition to the FNAH, we studied a case with SNAH (patient #121) and 2 children (patients #229 and #230) with overt hyperthyroidism; in one of those patients, we found a single nodule in the right lobe (patient #229), and we found multiple nodules and hemigoiter in the other patient (patient #230). Clinical characterization of these patients is described in the Supplemental Methods and Figure 2.

### Genetic screening for constitutively activating TSHR mutations in NAH.

In family A, a missense TSHR variant, c.710G>A, was detected in a heterozygous state in patient #270 and in all affected individuals, but not in 4 healthy family members from the same family (Figure 1A). The detected variant causes a substitution of serine (Ser) to asparagine (Asn) residue at position 237. The p.S237N mutation is located in exon 9 of the TSHR, which encodes extracellularly exposed parts in the ligand binding leucine-rich (Leu-rich) repeat domain (LRRD). In families B and C, heterozygous single nucleotide mutation c.1918A>G in the TSHR was identified in all affected cases (patients #266, #279, #280, #74, and #75). This mutation (p.I640V) leads to an amino acid change from isoleucine (Ile) to valine (Val) at position 640 (Figure 1B). In family D, all affected children had a previously described TSHR CAM c.1454C>T, leading to an Ala485Val (p.A485V) amino acid change (28). Furthermore, TSHR sequencing revealed another variant, c.697G>A in patient #293, leading to a Val233Met (p.V233M) amino acid change. However, this variant did not segregate and was predicted to be benign (Figure 1 and Supplemental Table 2).

In a patient with SNAH (patient #121), a heterozygous single nucleotide de novo mutation c.1887G>C in TSHR was identified with a leucine (Leu) to phenylalanine (Phe) amino acid exchange at position 629 (p.L629F; Figure 2) (29). In patient #229 with a single toxic nodule, heterozygous single-nucleotide mutation c.1897G>C in TSHR was found, leading to an Asp633His (p.D633H) amino acid change, as also previously described (23, 30). In contrast, patient #230 with a multinodular goiter was identified with a previously known single-nucleotide mutation c.1801T>A in TSHR, which leads to a Tyr601Asn (p.Y601N) variant (31). Of note, we did not find any other pathogenic or rare secondary findings in the exome coding areas in any of our patients with hyperthyroidism. However, 1 patient with a hot thyroid nodule (patient #230) carried a rare c.985C>T (p.Phe329Leu) PAX8 variant previously predicted as benign (Supplemental Table 2) (32).

### Screening of TSHR variants in FinnGen database.

To identify additional pathogenic TSHR variants at the population level, we screened the FinnGen project data release 11, which includes genotype and phenotype data from 473,681 individuals (33). As listed in Table 1, we identified 1 variant leading to loss of TSHR start codon (rs1305153461), 2 previously described inactivating variants (p.Cys390Trp and p.Arg531Gln), and 4 rare TSHR variants (p.Ile155Leu, p.Val424Phe, p.Arg531Gly, and p.Ile635Met). Although the more common variants Asp36His and Pro52Thr are often reported as clinically benign, a full phenotype scan of FinnGen shows that they are significantly associated to risk of hyperthyroidism but not with Mendelian penetrance. The variants rs1305153461 (variant leads to loss of the start codon) and p.Val424Phe were independently associated with increased OR for hypothyroidism (OR = 8.17, *P* = 2.6 × 10^–9^; OR = 5.4, *P* = 9.84 × 10^–7^) in the FinnGen database (Table 1). Functional screening of the variants identified from this database show no significant difference in basal cAMP production, although, for variant p.V424F, the basal cAMP concentrations were slightly decreased. However, a high-dose TSH stimulation (10 mU/mL) revealed reduced cAMP production with the TSHR variants p.Cys390Trp, p.Arg531Gly, and p.Val424Phe compared with the WT receptor (Supplemental Figure 4).

### TSHR variants associate with hypo- and hyperthyroidism but not with other phenotypes in FinnGen.

In addition to the primary role of TSHR in thyroid disorders, variations in normal thyroid function are associated with differences in body composition, glucose metabolism, and bone mineral density (34, 35). Therefore, we hypothesized that common variants at the TSHR locus could be associated with other phenotypes in the Finnish population. We screened the FinnGen database and found significant genome-wide associations exclusively between common TSHR variants and thyroid phenotypes within 100 kb of the TSHR. The strongest association was observed between thyrotoxicosis with diffuse goiter (lead variant rs1023586; *P* = 2.5×10^–51^), but only other endpoints related to hyperthyroidism were also significantly correlated (Supplemental Figure 5A). Detailed examination of this association signal indicated that the causal variant was likely one of a credible set of 4 common SNPs within a 30 kb segment of the long first intron of TSHR.

Independent of this association with thyrotoxicosis, a significant genome-wide association not previously reported was also observed with autoimmune hypothyroidism (lead variant rs12897126, *P* = 9.1 × 10^–17^). This association localized to 4 SNPs in a neighboring but nonoverlapping region of the TSHR intron 1 and was specifically associated with hypothyroid phenotypes (Supplemental Figure 5B). These 2 TSHR intron 1 associations were unrelated to each other by linkage disequilibrium (LD) (*r*^2^ < 0.05), and neither association showed any phenotypic crossover (i.e., the thyrotoxicosis association showed no relationship to hypothyroid risk and vice versa), suggesting potentially distinct mechanisms or context specificity underlying these 2 intronic associations. No other significant associations between the TSHR locus and the FinnGen disease endpoints unrelated to hypo- or hyperthyroidism were observed.

### TSHR variants p.I640V and p.S237N show increased constitutive activity in vitro.

**Figure 3:**
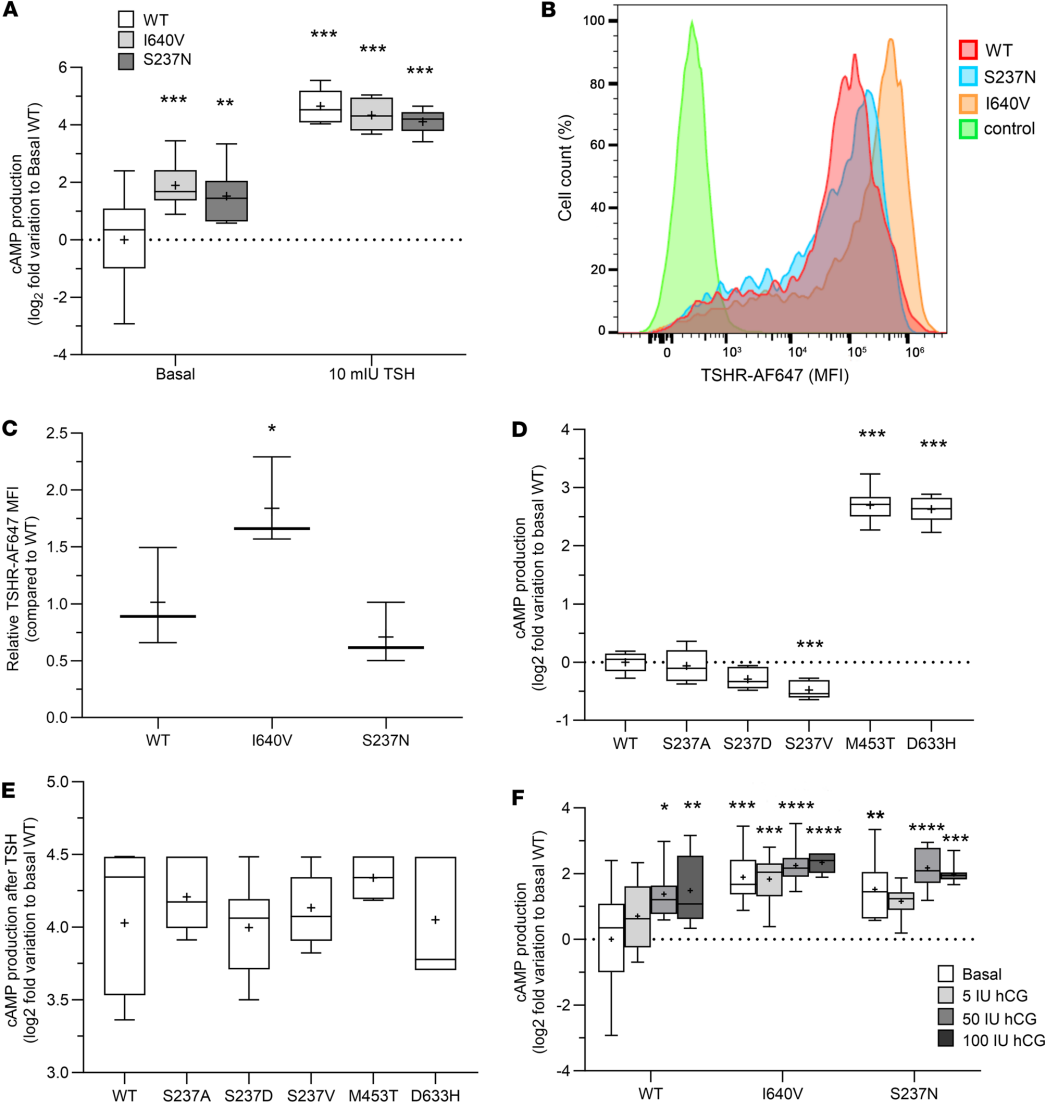
Functional characterization of the TSHR mutations in vitro. (**A**) Accumulation of cAMP in basal condition (constitutive activity) and after stimulation with high dose (10 mU/mL) of bovine TSH between TSHR WT, I640V, and S237N mutants using transiently transfected COS-7 cells. TSHR cell surface expression was assessed in live cells using flow cytometry (FC). (**B**) Representative FC profiles of COS-7 cells transfected with WT TSHR (red), S237N (blue), or I640V (orange) variants or negative control (green). (**C**) A graph with the relative median fluorescence intensity (MFI) of COS-7 cells expressing the TSHR WT or I640V and S237N mutants (*n* = 4). (**D** and **E**) Effect of different amino acid substitutions at TSHR position 237 (p.S237A, p.S237D, and p.S237V) on basal TSH-stimulated (10 mU/mL) cAMP compared with WT and previously described TSHR CAMs M453T and D633H mutants. (**F**) Production of cAMP in a response to different doses of human chorionic gonadotropin (hCG) using cells expressing TSHR WT, I640V, or S237N variants. Box-and-whisker plots show 95th–5th percentile, with median (line) and mean (+) symbols. The cAMP concentration were tested with 2-way ANOVA, whereas relative cell membrane TSHR expression was tested with 1-way ANOVA. Both tests were corrected for multiple hypothesis. The hCG-response assay was tested using univariate ANOVA. Family-wise error rate was controlled using Dunnett’s method, considering WT TSHR as the reference group in both conditions. Data are representative of 2 or 3 independent experiments. *P < 0.05; **P < 0.01; ***P < 0.001; ****P < 0.0001.

We tested the functionality of the TSHR variants p.I640V and p.S237N using heterologous cell assays. Constitutive activity of TSHR I640V and S237N mutants was increased slightly by 1.89-fold (SD ± 0.74) and 1.52-fold (SD ± 0.95), respectively, compared with the WT receptor, with similar responses to a high-dose TSH stimulation among all variants (Figure 3A). Transfection efficiency of WT and the mutants varied between 80% and 95% according to flow cytometry (FC) analysis. In live-cell FC tests, TSHR variant p.I640V showed the highest membrane expression per cell (1.84- ± 0.39-fold increase compared with WT receptor), whereas, in variant S237N, the median fluorescence intensity did not differ significantly from the WT (Figure 3, B and C). Next, the effect of different amino acid substitutions (p.S237A, p.S237D, and p.S237V) at TSHR position 237 on basal and stimulated cAMP production were compared against WT receptor, with TSHR CAMs p.M453T and p.D633H as positive controls (Figure 3, D and E). The basal signaling activity of the variant p.S237V was decreased (–0.48- ± 0.15-fold) when compared with the WT receptor, but the p.S237A and p.S237D mutations and a high TSH (10 mIU/mL) stimulus induced similar responses (Figure 3, D and E). Altogether, this suggests that the constitutive activation by p.S237N is rather specific for this side-chain variant because smaller hydrophobic (alanine), negatively charged hydrophilic (aspartate), or larger and bulkier (valine) side chains do not activate TSHR constitutively at this position.

To gain further molecular insights into the potential mechanism of TSHR constitutive activity, we tested the cAMP response of these mutations following a stimulation with human chorionic gonadotropin (hCG; 5, 50, 100 IU) as has been reported in some cases of temporary hyperthyroidism (36). A significant difference was observed in the dose-response curve as a function of the TSHR variant (F_[2,__84]_ = 16.24, *P* < 0.0001) and hCG dose (F_[3,__84]_ = 6.280, *P* = 0.0007), but not as a function of the interaction variant dose (Figure 3F). The p.I640V and p.S237N variants showed increased cAMP secretion as hCG doses increased, but they already had high basal cAMP production.

### TSHR-mediated NAH phenotype depends on mutation properties and dietary iodine content.

**Figure 4:**
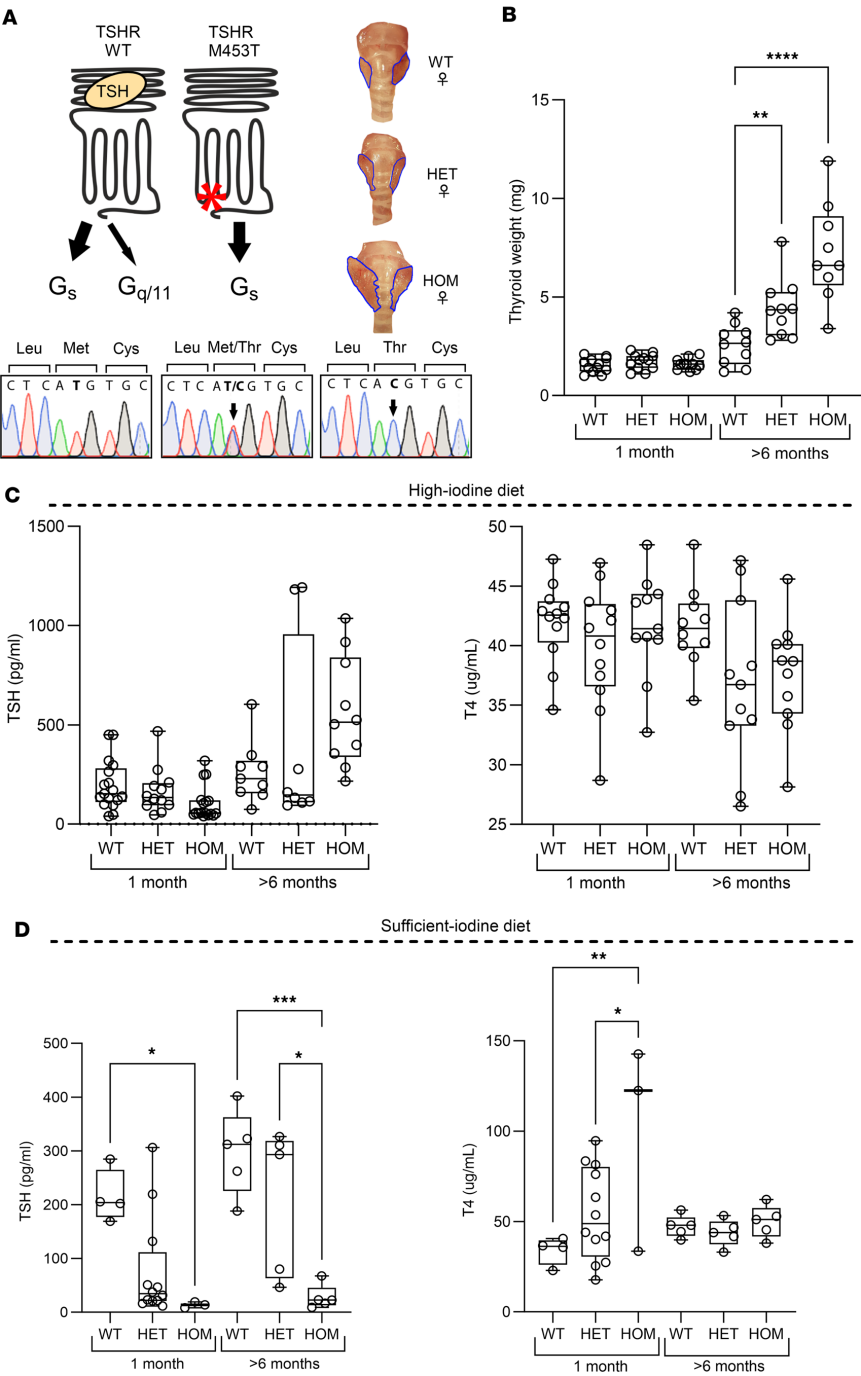
Generation and characterization of the thyroid phenotype in knockin mice carrying a constitutively active TSHR M453T mutation. (**A**) Illustration of the location of TSHR M453T mutation and its G protein signaling preferences compared with WT TSHR. Below is a representative genotyping chromatogram of WT, heterozygous (HET), and homozygous (HOM) TSHR M453T mice, from left to right. The generation of the model using CRISPR/Cas9 and homology-directed repair technique, genomic location, and designed guide RNA (gRNA) construct and genotyping protocol is described in Supplemental Figure 7. On the right, representative thyroid images of WT, HET, and HOM TSHR female mice. (**B**–**D**) Thyroid weight (**B**) and serum TSH and T4 concentrations of 1 and >6-month-old WT, HET, and HOM TSHR mutant mice under high-iodine (**C**) and sufficient-iodine (**D**) diets. *n* = 3–17 male and female mice per genotype. Box-and-whisker plot represents median, 25th, and 75th percentile, with whiskers from minimum to maximum with all points shown. Statistical analysis was carried out using the 1-way ANOVA with Bonferroni’s multiple-comparisons test. *P < 0.05; **P < 0.01; ***P < 0.001; ****P < 0.0001.

To study the in vivo effect of altered TSHR signaling, we generated and compared the phenotype of TSHR M453T mutant mice with our previously generated D633H model (23). As illustrated in Figure 4A, the TSHR M453T variant leads to strong cAMP/Gα_s_ activation in vitro, while D633H activates both Gα_s_ and Gα_q_ cascades (23, 37). Successful insertion of the M453T mutation (c.1358T>C) into the murine Tshr locus using the CRISPR/Cas9 technique was confirmed by PCR with restriction enzyme digestion and sequencing of the founder’s genomic DNA (Figure 4A and Supplemental Figure 6).

Similar to the D633H model (23), the thyroid weight was significantly increased in > 6-month-old heterozygous and homozygous TSHR M453T mice when compared with WT littermates (Figure 4, A and B). Furthermore, homozygous TSHR M453T mice showed slightly reduced serum TSH but normal T4 at 1 month of age under a standard mouse chow diet containing an excess (6 mg/kg) of iodine, when compared with WT littermates. Unexpectedly, still under the iodine excess diet, serum TSH concentration was significantly increased in several older (>6-month-old) M453T mutant mice compared with WT controls (Figure 4C). No significant differences were found in serum T4 concentrations between WT and mutants of any genotype at 1 or 6 months of age (Figure 4C) under a high-iodine diet. Interestingly, when the diets of 1- or >6-month-old mice were replaced with a sufficient-iodine diet (0.3 mg/kg) for 1 month, the serum TSH concentrations of homozygous M453T mice were significantly lower, and T4 concentrations were higher, than those of mutants under standard/high-iodine diet (Figure 4D).

**Figure 5:**
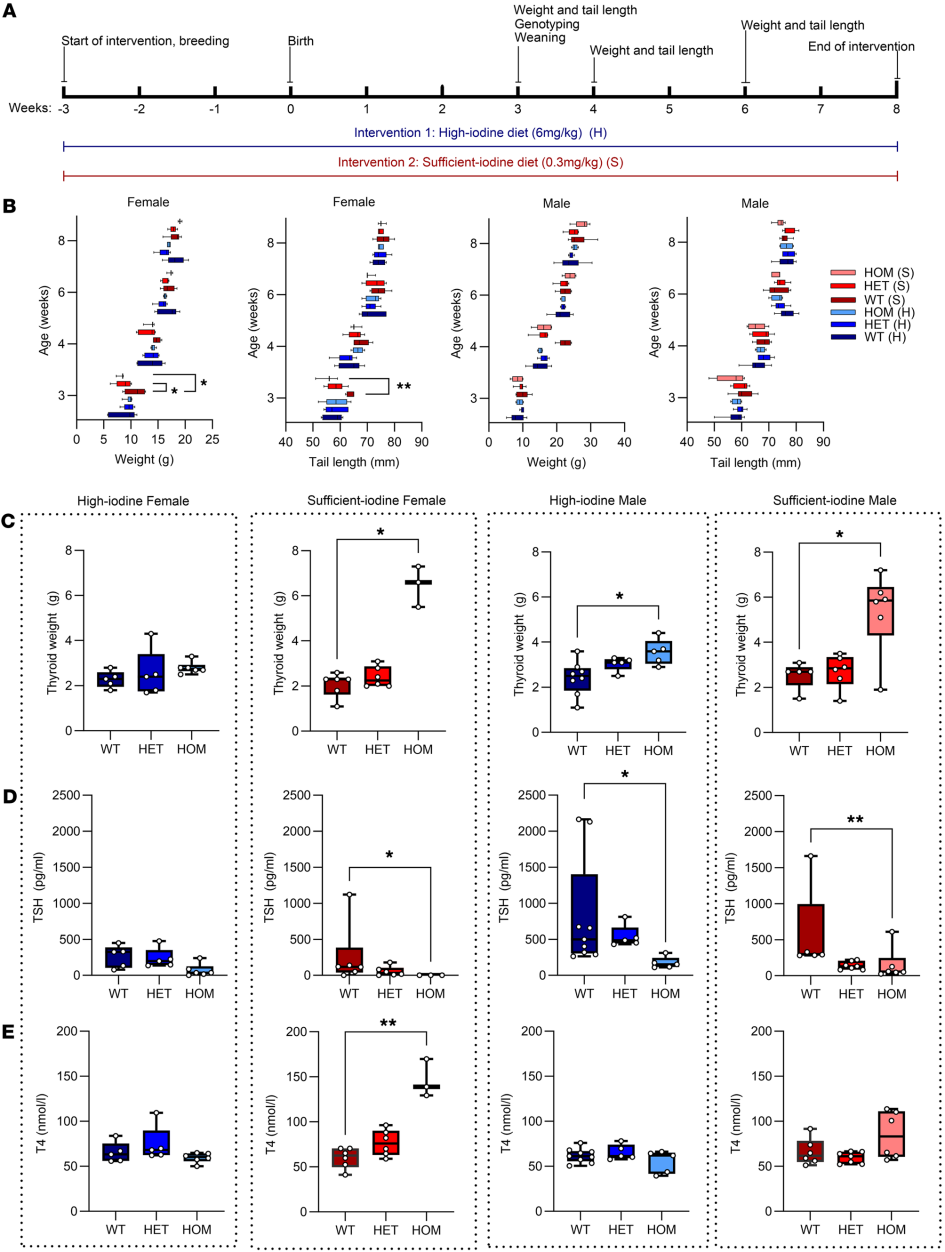
Effect of dietary iodine on the phenotype of TSHR knockin mice carrying a constitutively active TSHR M453T mutation. (**A**) Illustration of the experimental set up. (**B**) Weight and tail length of the 3- to 8-week-old WT, heterozygous (HET), and homozygous (HOM) TSHR mutant mice under high-iodine (H) and sufficient-iodine (S) diets. (**C**–**E**) The thyroid weight, serum TSH, and total T4 concentrations at the end of 8-week follow-up. *n* = 3–9 male and female mice per genotype. Box-and-whisker plots represent median, 25th and 75th percentile, with whiskers from minimum to maximum with all points shown, except for **B**. Statistical analysis was carried out using the one-way ANOVA with Šidák multiple-comparison test in **B** and Kruskal-Wallis test with Dunn’s multiple-comparison test in **C**–**E**. *P < 0.05; **P < 0.01.

To further evaluate the effect of dietary iodine on the NAH phenotype, we compared the effect of an 8-week treatment with a high-iodine diet (6 mg/kg) to an otherwise identical diet with a sufficient amount of iodine (0.33 mg/kg). We assessed body weight, tail length, thyroid weight, and thyroid function in both sexes (Figure 5A). As shown in Figure 5B, the homozygous M453T females treated with a sufficient-iodine diet had lower body weight and shorter tail length when compared with the WT littermates at the age of weaning (3 weeks). Homozygous TSHR M453T mice also gained weight more rapidly during the 8-week treatment when fed with sufficient-iodine diet compared with high-iodine diet. No difference in weight gain or tail growth was seen in TSHR M453T mice under high-iodine diet when compared with WT controls. Most obvious findings were observed in thyroid weight (Figure 5C). In homozygous TSHR M453T males and females receiving sufficient-iodine diet, the thyroid weight at 8 weeks of age was significantly increased when compared with WTs. In contrast, in homozygous females receiving a high-iodine diet, the thyroids were not significantly larger than those of the WTs, and in homozygous males, the increase was less pronounced. The changes in thyroid weight, body weight, and tail length during the 8-week treatment were clearly associated with the development of hyperthyroidism (low serum TSH, high T4), most notable in homozygous TSHR M453T females in sufficient-iodine group (Figure 5, D and E).

**Figure 6:**
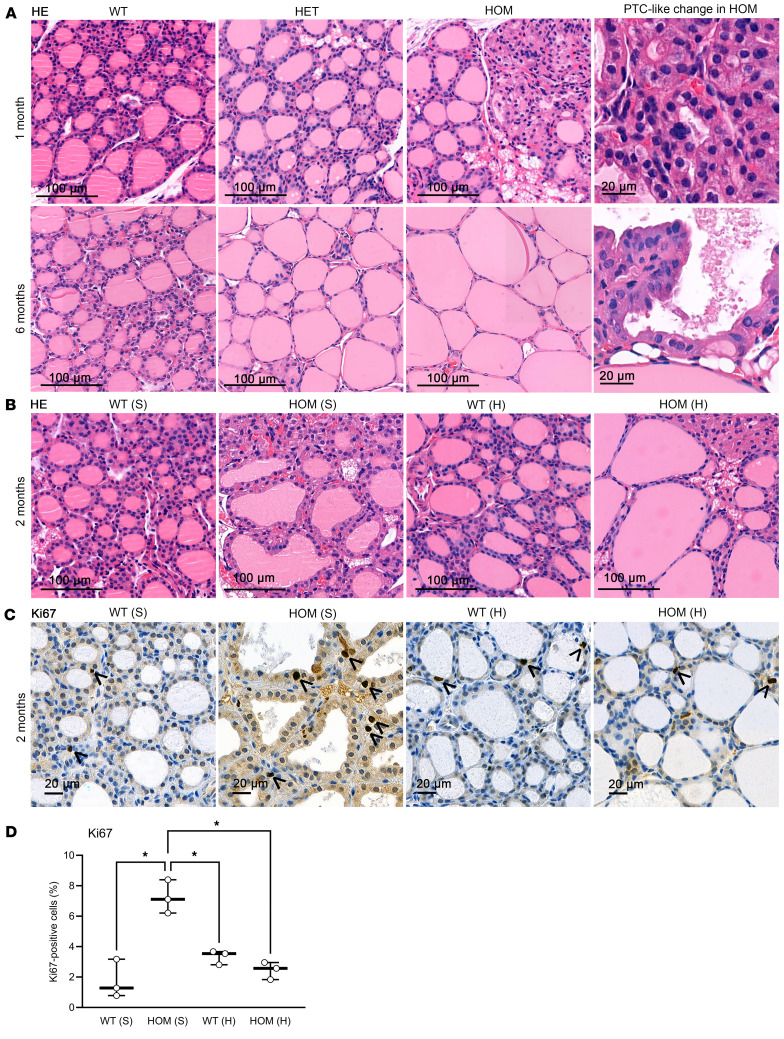
Thyroid histology of knockin mice carrying a constitutively active TSHR M453T mutation. (**A**) Representative H&E-stained thyroid images of WT, heterozygous (HET), and homozygous (HOM) female mice at 1 and 6 months of age. Scale bars: 100 μm. On the right, 2 magnifications of papillary thyroid carcinoma–like areas in HOM thyroid. Scale bars: 20 μm. (**B**) Representative H&E-stained images of thyroid tissue of WT and HOM mice after 2 months sufficient-iodine (S) or high-iodine (H) diets. Scale bars: 100 μm. (**C**) Ki67 IHC of thyroid sections of WT and HOM female mice, born and maintained under S or H diet for 2 months. Scale bars: 20 μm. Arrowheads indicate Ki67-stained nuclei. (**D**) The number of Ki67^+^ cells relative to the total number of thyrocytes of 2-month-old female WT and HOM mice that uphold their lifetime with S or H diets (*n* = 3–5 mice/group). Box-and-whisker plot with median, 25th and 75th percentiles shown with box, and minimum to maximum with whiskers. Individual samples shown as circles. Brown-Forsythe and Welch 1-way ANOVA tests with Dunn’s multiple comparison. *P < 0.05.

Histologically, under a high-iodine diet, the heterozygous and homozygous mice at 1 month of age presented with slightly smaller follicles, thicker layers of thyrocytes, more vacuoles, and occasionally hypertrophied areas of diminished colloid compared with WT mice. However, prolonged exposure (>2 months) to high-iodine diet led to the development of colloid goiter with large follicles and a thin thyroid epithelium in the homozygous TSHR M453T mice, whereas, under sufficient-iodine diet, their thyroid histology showed features seen typically in hyperthyroidism and high TSH stimulation (hyperplasia, protrusion, reduced amount of colloid). Furthermore, in a few 1-month-old and several >6-month-old homozygous M453T mice, large hyperplastic thyroid areas with PTC-like changes were seen (Figure 6).

Two-month-old homozygous TSHR M453T female mice under a sufficient-iodine diet had significantly increased proliferation index (Ki67^+^ cells) compared with WT thyroids (7.2% ± 1.1% in mutants versus 1.7% ± 1.3% in WTs; Figure 6). Switching the diet to high-iodine decreased the proliferation rate of the mutants to the level of WTs (2.5% ± 0.6% in mutants versus 3.3% ± 0.5 % in WT thyroids).

**Figure 7:**
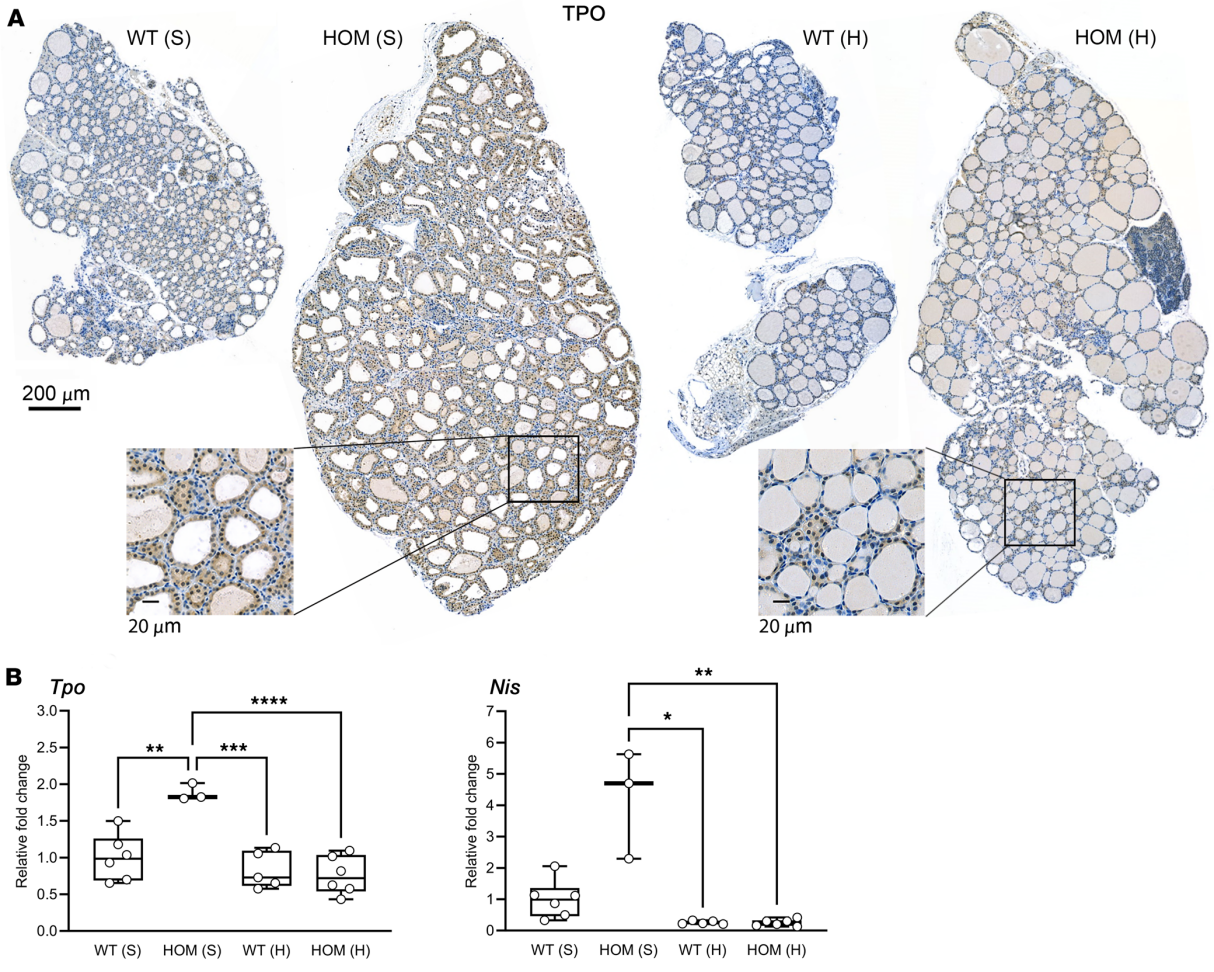
*Tpo* and *Nis* expression in thyroids of WT and TSHR M453T HOM mice after 2 months of S and H diets. (**A**) Representative TPO IHC data. Scale bars: 200 μm and 20 μm (insets). (**B**) Relative gene expression of *Tpo* and *Nis* in thyroids, quantified by qPCR. The data are presented as fold change in HOM S (*n* = 3), WT H (*n* = 5), and HOM H (*n* = 6) relative to WT S (*n* = 6) as the calibrator. Expressed as mean ± SD; statistical significance for Tpo (1-way ANOVA, Tukey’s test) and for Nis (Kruskal-Wallis, Dunn’s test). *P < 0.05, **P < 0.01, ***P < 0.001, ****P < 0.0001. Boxes show median, 25th and 75th percentiles; whiskers show minimum to maximum.

Next, we tested the effect of the TSHR CAM and dietary iodine concentration on sodium-iodide symporter (Nis) and thyroid peroxidase (TPo) expression using quantitative PCR (qPCR) and IHC. As shown in Figure 7, more abundant Tpo staining were detected in 2-month-old mutant mice under sufficient-iodine diet compared with corresponding high-iodine groups. In the qPCR analysis, upregulation of *Tpo* and *Nis* expression was observed in TSHR mutant mice with a sufficient-iodine diet compared with WTs, whereas exposure to a high-iodine diet reduced their expression, most notably as a decrease in *Nis* gene expression.

### TSHR variants in light of TSHR structures.

Recently, diverse TSHR structures in different activity state–associated conformations were determined by cryogenic electron microscopy (cryo-EM) methods (38, 39). The inactive state of the TSHR WT structure (PDB ID 7t9m) was used to highlight the positions of the TSHR variants identified in this study (Supplemental Figure 8 and Supplemental Methods). The intracellularly located variant p.R531G likely modulates the G protein/receptor interplay, as previously described for the mutation p.R531Q (40) related to hypothyroidism. Variant p.V424F affects the receptor fold and/or signal transduction mechanism because of sterical conflicts in the interhelical (between transmembrane helix [TMH] 1 and 7) packing by this substitution toward a bulkier and longer side chain of Phe. Mutation p.C390W at cysteine (Supplemental Figure 8A) is essential for constituting disulfide-bridges and has a negative effect on receptor folding and expression as shown previously (41).

To understand the details of the molecular mechanisms of the identified TSHR CAMs, the active state complex with bound TSH and Gα_s_ at the TSHR (PDB ID 7t9i; ref. 38) (Supplemental Figure 8B) can be studied. The identified known mutations p.Y601N, p.L629F, p.D633H, and the potentially novel p.I640V variant are all located in transmembrane spanning helices 5 and 6, at spatial receptor regions strongly involved in signal transduction. Mutations at these positions destabilize the inactive state kept by WT side chain interactions to neighboring helices (Supplemental Figure 8, B–D). As a result, the TMH6 N-terminus moves strongly outward compared with the inactive state conformation, which is a known and common feature in GPCR activation.

The mutation p.S237N is specifically interesting, since it is located at the extracellular Leu rich repeat 9 (LRR9), whereby the entire LRRD is constituted by 11 repeats (amino acids Cys24-Asn288; Supplemental Figure 8C). Moreover, Ser237 is not in the hormone binding site and not in direct contact with any other side chain in the LRRD or in the transmembrane domain (TMD). Of note, further side chain variations p.S237A, p.S237D, and p.S237V did not result in constitutive activation, and this TSHR CAM cannot be hyperstimulated by an increased affinity of CG to TSHR (Figure 3D).This supports a specific activation mechanism for variant p.S237N.

## Discussion

Thyrotropin receptor–mediated (TSHR-mediated) diseases vary from loss-of-function mutations, which cause congenital hypothyroidism, to gain-of-function mutations, the primary etiologies for NAH. Although CAMs are rare and have been described only in approximately 40 families to date (10), the identification of the underlying mutations for NAH is crucial. Hyperthyroidism in these patients is usually permanent, and ablative treatment is recommended. However, the precise mechanisms underlying the development of clinical hyperthyroidism and toxic thyroid nodules due to TSHR gain-of-function mutations is not comprehensively understood. Other genetic factors or dietary iodine concentration have been suggested to play a role as modifying factors for disease development. Here, we described and characterized TSHR mutations in a broad spectrum of thyroid phenotypes, including 4 FNAH cases (with TSHR p.S237N and p.I640V CAMs), 1 SNAH with de novo germline TSHR mutation (p.L629F), 2 TSHR CAMs (p.D633H and p.Y601N) identified in children with toxic single nodules and multinodular goiter, and several TSHR loss-of-function mutations with association to hypothyroidism. Of note, our results reveal that, besides mutations in TSHR at position S281 (42, 43), further mutations in the extracellular LRRD of TSHR can trigger constitutive signaling activity. This activity is neither linked to hypersensitivity to other glycoprotein hormones (36) nor to direct activation of the internal TSHR agonist (44). Moreover, we show that TSHR-mediated phenotype depends on TSHR mutant signaling and dietary iodine content in a rodent model carrying a human-derived TSHR M453T mutation.

### Molecular details and mechanisms of newly identified TSHR CAMs.

Most TSHR CAMs are located in the TMD (constituted by the membrane-spanning helices and interconnecting loops), with the highest frequency in the sixth TMH (3). Interestingly, in this study, we identified a mutation leading to Ser-to-Asn conversion at extracellular position 237 in a family with NAH across 3 generations. Although the Ser at position 237 is not conserved among other glycoprotein hormone receptor homologs, its absence from population databases such as gnomAD and from the unaffected family members supports the variant’s pathogenic role.

Moreover, functional tests revealed that TSHR p.S237N leads to constitutive signaling activity (Figure 3A). Other CAMs in the N-terminal extracellular region of the receptor are rare. Naturally occurring mutations at position 281 have been reported in patients with toxic adenomas and have been shown to trigger TSHR constitutive activity (42, 43). The Ser281 is located in the transition between the LRRD and the hinge region, in a pivotal helix of repeat 11, which is crucial in regulating TSHR constitutive activity via interrelating the ligand binding and TMDs, whereas an activating mutation at position 237 is unexpected based on the recently published TSHR structures and derived functional models (38, 45, 46). In contrast to Ser281, Ser237 is not in any spatial proximity to intra- or interrelated interaction partners. Of note, the p.S237N substitution would add a new glycosylation site at the LRRD of TSHR, which is also supported by the adjacent occurrence of a threonine (Thr239), altogether constituting an Asn-linked glycosylation motif (47). Glycosylations can be diverse in their molecular constitution, also dependent on the TSHR-expressing cell type. In any case, glycosylations have a strong effect on receptor (and hormone) properties and functionalities (47–49). We hypothesize that a new glycosylation at this LRRD may modulate the basal signaling activity by affecting the structural adjustment and dynamic properties of the N-terminal receptor part, linked directly with signaling activity. The fact that the other side-chain substitutions at position 237 tested here (p.S237A, p.S237D, p.S237V) did not result in constitutive activation supports the idea of a new and alternative mechanism for constitutive activation at this position of the TSHR.

The naturally occurring TSHR p.I640V mutation has not been reported, but a mutation in the same position (p.I640K) has been described in a patient with a hot thyroid nodule and increased (up to 5.9-fold) basal cAMP activity compared with the WT receptor (50). Furthermore, the functionality of p.I640V was tested in vitro in another report that investigated the molecular mechanisms of constitutive activity. The replacement of Ile at position 640 with Val induces constitutive activity with 2.4 times higher basal cAMP production than the WT receptor (Figure 3A; ref. 51). As visualized in Supplemental Figure 8D and described in more detail in Supplemental Methods, Ile640 is located in an essential position between TMH6 and TMH7 and stabilizes the inactive state conformation by tight hydrophobic interactions. The shorter and bulkier side chain of Val due to the p.I640V substitution cannot maintain the surrounding lock-like interaction core in an inactive state, and this leads to constitutive receptor activation by a release of TMH6 and subsequent G protein coupling.

### Development of NAH and its activation during pregnancy among individuals with TSHR p.I640V mutation.

In contrast to the increased TSHR I640V in vitro basal signaling activity, the hyperthyroid phenotype was relatively mild and initially subclinical among all our study participants. However, all 3 individuals with the p.I640V mutation in family B had persisting hyperthyroid symptoms and required additional antithyroid medication, thyroidectomy, or radioactive iodine treatment. In contrast, 2 individuals (Family C) with the same mutation had subclinical hyperthyroidism (over 10 and 20 years) and did not require any treatment due to lack of hyperthyroid symptoms, goiter, osteoporosis, occurrence of atrial fibrillations, or other extrathyroidal manifestations. Interestingly, among the 2 family members with TSHR I640V, hyperthyroidism was activated in the early phase of pregnancy; thus, antithyroid medication was initiated. In line with clinical observation, hCG was able to further stimulate the activity of TSHR p.I640V, similarly as described recently with p.V597I mutation (52). In general, familial gestational hyperthyroidism caused by TSHR CAM is rare. So far, only 4 mutations, 2 in the ligand-binding LRRD and 1 in the TMD, have been reported (36, 53, 54).

### Role of TSHR CAM in vitro activity and genetic factors in NAH phenotype variability.

Currently, there is no clear evidence for the association between in vitro activity (usually tested in nonthyroidal COS-7 cells) and the severity of hyperthyroidism. Therefore, different environmental and genetic factors that modify the constitutive activity of the receptor or phenotype manifestation have been proposed. Differences in receptor posttranslational processing and glycosylation activity could be more obvious when tested in thyroidal cells and could explain some of the variation (55). Other genetic variations have been suggested to cause phenotypic variability among patients with TSHR CAMs. For instance, genetic variants of other signaling molecules such as phosphodiesterase, β-arrestin, or adenylate cyclase may attenuate or exacerbate hyperthyroidism (56). Genome-wide association studies (GWAS) associate genetic variants related to TSH and T4 secretion and release or mutations in TSHR loci with the severity of the disease (57–59). In our study, using exome sequencing, a panel of thyroid-related genes were screened but, apart from rare variants in TG (Asp2318Asn), PAX8 (Phe329Leu), and TSHR (Val233Met), no additional mutations were detected.

### TSHR variants associate with hypo- and hyperthyroidism but not with other phenotypes in FinnGen.

Using the longitudinal data in the FinnGen database, we conducted an extensive evaluation to investigate whether additional variants within the TSHR locus are associated with thyroid or nonthyroid phenotypes. Previous reports have suggested potential extrathyroidal expressions of TSHR in bone and adipose tissue, as well as associations between TSHR polymorphisms and factors like glucose metabolism and bone mineral density in selected studies (34, 35), albeit with inconsistent results (60).

In our study, we identified significant genome-wide associations exclusively between common TSHR variants and thyroid-related phenotypes, particularly those within 100 kb of the TSHR gene. However, no significant associations were observed between the TSHR locus and the >2,000 disease endpoints encompassed by the FinnGen database. While we acknowledge that definitive conclusions cannot be drawn from these findings alone, they suggest that the reported TSH/TSHR extrathyroidal associations may be more likely mediated through thyroid hormones.

Of particular interest, we observed the strongest associations in the FinnGen population between 2 unrelated variants situated in the first intron of TSHR, with 1 variant strongly associated with thyrotoxicosis and the other with autoimmune hypothyroidism. Notably, these 2 intronic TSHR associations were found to be unrelated to each other based on LD analysis. Furthermore, neither association demonstrated any phenotypic crossover, implying potentially distinct mechanisms or context specificity underlying these 2 intronic associations. Our results support the functional role of the intronic TSHR variants, which have been suggested to play a role in modulating central tolerance through influencing the intrathymic expression of TSHR (61).

### NAH phenotype in mice varies between different CAMs and dietary iodine content.

In our screening, 2 somatic TSHR mutations (D633H and Y601N) were identified in children with toxic nodules. Interestingly, the mutation D633H was the same as that in our recently generated knockin mouse model carrying this patient-derived TSHR mutation (23). Our results from this model indicate that NAH is a dynamic condition involving age, sex, and TSHR allele-dependent compensatory mechanisms. Furthermore, thyroid histology in this model changes from mild hypertrophy at an early age to hypothyroid-like follicles, including formation of colloid goiter before the development of papillary thyroid cancer–like features at an older age. Here, we compared the phenotype of TSHR M453T knockin to that previously described D633H model to elucidate whether the strong Gα_s_-mediated basal activity in TSHR M453T mutant differs from that of D633H, which activates both Gα_s_ - and Gα_q/11_-signaling pathways. Interestingly, initial analysis using TSHR M453T mice under a standard chow diet with a relatively high iodine (6 mg/kg) content led to a similar phenotype as described in the D633H model (23). However, during 4–8 weeks of sufficient-iodine (0.3 mg/kg) diet, the homozygous TSHR M453T mice developed hyperthyroidism, associated with goiter, hypertrophic thyroid follicles, and increased thyrocyte proliferation. This suggests that prolonged high iodine exposure inhibits thyroid function in this model carrying a TSHR CAM with Gα_s_/cAMP basal activity. We did not observe a similar effect in TSHR D633H–knockin mice, indicating that the effect is mutation or signaling specific and that the constitutive active Gα_q/11_-mediated signaling in this model appears to affect this phenomenon. In humans, prolonged iodine supplementation ameliorating hyperthyroidism has been reported in rare cases of hyperthyroid patients with strong Gα_s_-biased mutations (62). This could be due to the differences in TSHR CAM signaling on iodine-mediated sodium-iodine symporter (NIS) or TPO expression, as it has been shown that acute or chronic exposure to iodide excess can inhibit NIS and TPO expression, and this could affect the escape from the acute Wolff-Chaikoff effect (63). Similarly, in homozygous TSHR M453T, a high-iodine diet reduced the expression of Tpo and Nis, and the reduction was more apparent in *Nis* mRNA when compared with the homozygous or WT models receiving sufficient-iodine diet. However, further studies are required to evaluate more detailed mechanisms of the inhibitory effects of high-iodine diet on these TSHR CAM models.

### TSHR loss-of-function mutations and wide spectrum of hypothyroidism phenotype.

Loss-of-function mutations in TSHR lead to variable degrees of TSH resistance, presenting with severe CH to subclinical hypothyroidism with mildly elevated TSH. To date, the largest long-term follow-up of patients with TSHR loss-of-function mutations showed a trend toward increased TSH and decreased fT4 over time in homozygous patients, while fT4 levels remained stable in heterozygous carriers, thus giving a perspective on treatment strategies for these patients (64). However, considering the wide phenotypic variability, replacement therapy and follow-up strategies should be designed on an individual basis. In our screening of patients in the FinnGen database with nonautoimmune congenital hypothyroidism, TSH resistance, or rare TSHR variants linked to hypothyroidism, we identified both what we believe to be novel and previously known TSHR mutations. Additionally, variants outside the TSHR coding region, along with oligogenic variants as recently reported in various CH cohorts (65), collectively contribute to the observed phenotypic variation.

In summary, here we report potentially new coding and noncoding genetic variants in TSHR locus associated with TSHR-mediated diseases, but we report no other association between TSHR loci and over 2,000 disease endpoints in the well-powered FinnGen database. Furthermore, we describe TSHR CAMs in families with NAH, among them a TSHR S237N mutation in an atypical location in the extracellular domain, suggesting a potentially new mechanism for intrinsic activation of the TSHR. Functional characterization of the TSHR M453T–knockin mouse model revealed that the presence of the TSHR CAM phenotype depends on the signaling properties of the mutation and can be ameliorated by increased iodine intake. Finally, our data suggest that TSHR-mediated disease risk can be modified by variants in the TSHR locus both inside and outside the coding region as well as by altered TSHR-signaling and dietary iodine, supporting the need for personalized treatment strategies.

## Methods

### An association study between TSHR variants and phenotypes.

The FinnGen data release 11 was used in this study. Detailed information about the different data releases is described in FinnGen’s website (33). For the phenotype analysis, as a part of the FinnGen project, genetic data from Finnish participants was linked with disease endpoints (66) constructed from Finnish national hospital registries and cause-of-death registry using International Classification of Diseases (ICD), Social Insurance Institute (Kela) drug reimbursement, and Anatomical Therapeutic Chemical (ATC) codes. Data release 11 includes 473,681 Finnish individuals and contains numerous cases with thyroid diseases (for example, 459 individuals with congenital hypothyroidism; 54,752 with hypothyroidism; and 2,469 cases of autoimmune hyperthyroidism). More details on the phenotypes are available at the FinnGen website (67).

### Thyroid function tests, thyroid imaging, and IHC.

The P-TSH and fT4 levels were measured using standard chemiluminometric assays (Abbott Architect) used at the University Hospital laboratories in Finland (Turku, Oulu, Helsinki, and Kuopio). The detection limit for P-TSH was 0.03 mU/L, and the TSH and fT4 age-dependent reference ranges varied from 0.1–17 mU/L and 10–36 pmol/L in 1- to 3-day-old newborns to 0.5–4.0 mU/L and 9.0–19 pmol/L in adults, respectively. Ultrasound imaging was performed by pediatric radiologists. For the family of Polish origin, the plasma levels of fT4, fT3, TSH, and serum anti-TPO, anti-TG, and TSHR-antibodies were determined on electrochemiluminescence immunoassay (ECLIA) with a cobas e 411 analyzer (Roche Diagnostics). Normal values for fT4 ranged between 1.1 and 1.7 ng/dL, for fT3 between 2.3 and 5.0 pg/mL and for TSH between 0.28 and 4.3 mU/L, and the cut-off for thyroid antibody tests were: 1.7 U/L for anti-TSHR antibodies, between 0 and 34 IU/mL for anti-TPO antibodies, and between 0 and 115 IU/mL for anti-TG antibodies. The iodine uptake test was performed by i.v. administration of 0.3 mCi (equivalent to 11.1 MBq) Technetium-99m (^99m^Tc) followed by measurement of the uptake above the thyroid after 30 minutes from application. H&E staining was performed using 4 μm–thick FFPE sections, deparaffinized, and stained according to the standard protocols (22), with antibodies listed in Supplemental Table 5.

### NGS screening, PCR, and Sanger sequencing of the TSHR mutations.

DNA for the genomic testing was isolated from the leukocytes of peripheral blood or from paraffin-embedded thyroid tissues from patients with thyroid nodules using commercial kits (Qiagen and Nucleospin DNA FFPE kit, Macherey-Nagel). The DNA from the index patients was sequenced with next-generation sequencing (NGS) panel as described previously (35). All the mutations detected by NGS method were first visually evaluated using Integrative Genomics Viewer (68) (Supplemental Figure 6) and then confirmed by Sanger sequencing using a commercial service (GATC Biotech). PCR for Sanger sequencing was performed using following conditions: 150 ng of genomic DNA template was added to a 25 μL reaction mix (1× DyNAzyme buffer [Thermo Fisher Scientific], 0.2 nmol/L dNTP, 1 μmol/L of each primer, 0.02 U/μM DyNAzyme polymerase [Thermo Fisher Scientific], and Molecular Biology grade water [B. Braun Melsungen AG]). The primer pairs are listed in Supplemental Table 3. The PCR was performed in a thermocycler (Biometra) with following program: initial denaturation (2 minutes, 94°C); 35 cycles of denaturation (30 seconds, 94°C), annealing (30 seconds, 63°C–65°C according to the primer pair), and elongation (1 minute, 72°C); and a final elongation (10 minutes, 72°C). Afterward, the PCR product was run on 2 % agarose gel, cut, and purified using NucleoSpin Gel and PCR Clean-up kit (Macherey-Nagel).

### Generation of the TSHR mutant vectors and testing the constitutive activity.

All TSHR mutants were generated using commercial DNA synthesis service (BioCat). The template vector used was pcDNA3.1Zeo(-) WT TSHR with HA-tag (NM_00369.5) as described in a previous publication (31). The constitutive activity of WT and mutant receptors was tested using COS-7 cells (from Jukka Kero’s Lab, Department of Clinical Sciences, University of Turku) grown in DMEM and Ham’s F12 nutrient mixture (DMEM/F12, Sigma-Aldrich) supplemented with 10% FBS (Sigma-Aldrich), 105U/L penicillin, and 100 mg/L streptomycin (Sigma-Aldrich) at 37°C in a humified 5% CO_2_ incubator. Before transfection, 100,000 cells were plated on 96-well plate and then transfected with 200 ng DNA per well using DharmaFECT kb DNA transfection reagent (Horizon Discovery) according to the manufacturer’s protocol. For the cAMP assay, 48 hours after transfection, the cells were first starved for 1 hour in serum-free DMEM/F12 medium and then incubated for 1 hour with 0.1 mM 3-isobutyl-1-methylxanthine (IBMX; Sigma-Aldrich) with or without stimulating ligands (10 mIU/mL of recombinant TSH or 5, 50, or 100 IU/mL of hCG; Sigma-Aldrich) diluted in serum-free medium. The medium was then collected for cAMP ELISA assay (Cayman Chemical) performed according to the manufacturer’s instructions.

### FC.

TSHR cell membrane expression in live cells was quantified using previously described protocol (69). Briefly, 200 ng of plasmid DNA containing WT or mutant TSHR were transfected as described above. Transfected cells were detached from the dishes using 1 mL Accutase 400–600 units/mL (Sigma-Aldrich) per well before being dispensed in 2% v/v FBS/PBS buffer. The analysis was performed using ACEA Novocyte flow cytometer (ACEA Biosciences Inc.) with following antibody dilutions: 1:50 dilution of rabbit anti–human HA-tag IgG (Novus Biologicals), 1:100 dilution of Alexa Fluor 488–labeled goat anti–rabbit IgG (Invitrogen, Molecular Probes), and 1:100 dilution of Alexa Fluor 647–labeled goat anti–rabbit IgG (Invitrogen, Molecular Probes) in FACS buffer (BD Life Sciences). The antibodies are listed in Supplemental Table 5. Membrane TSHR expression was determined by the median fluorescence intensity using FlowJo v10.8 Software (BD Life Sciences).

### Generation and analysis of TSHR M453T–knockin mouse model.

Knock–in mice carrying a previously described human-derived TSHR M453T point mutation (70) were generated using CRISPR/Cas9 and homology-directed repair technique as described earlier (71). Briefly, following guide RNA (sgRNA) and single-strained DNA (ssDNA) templates were used; sgRNA: 5′-CAAAGGCCAAGTTGCACATGAGG-3′ and ssDNA: 5′-CATTCTGCTAACCAGCCACTACAAATTGACCGTGCCGCGGTTCCTCACGTGCAACTTGGCCTTTGCAGATTTCTGCATGGGGGTATACCTGCTTC-3′, designed to target exon 10 of the TSHR. Cas9 mRNA and sgRNA were introduced into zygotes by electroporation. The correct mutation was confirmed with Sanger sequencing (Eurofins Genomics) and digestion of the PCR product with restriction enzymes. The following primers were used in genotyping: forward, 5′-AATCGTGGTGTGGTTTGTCA-3′; reverse, 5′-CCTACCGGAATTCGCGTCTCGAGAGGGTGATGACCGTCAGTGT-3′. Experiments were performed in mixed genetic background of C57BL6/J and C57BL6/Ncr using WT littermates as controls. The experimental groups were generated by heterozygous breeding, and this resulted in expected distribution of the genotypes, sex (1:1.1), and normal litter size (mean, 6.8 pups; 2.30 SD). Mice were housed under controlled conditions (individually ventilated cages, 12-hour light/12-hour dark, 21 ± 1°C) at the Central Animal Laboratory, University of Turku. For maintenance and for the experiment presented in Figure 4, the animals were provided ad libitum access to water and pelleted chow containing high-iodine diet (6 mg/kg, Teklad 2018 global 18% protein chow, Envigo RMS Division) or sufficient-iodine diet (0.3 mg/kg, Ssniff’s AIN 93M chow, Ssniff). For the experiment presented in the Figure 5, high-iodine diet (Envigo, Teklad, TD.230152, supplemented with potassium iodate to reach 6 mg/kg iodine concentration in the final chow) was compared with the otherwise identical diet containing sufficient amount of iodine (Envigo, TD.230025, 0.33 mg/kg iodine). These diets were a modification of the iodine-deficient diet (TD.120363).

Animals were sacrificed with CO_2_, and blood was collected via cardiac puncture. Serum total T4 and TSH concentrations were quantified similarly as previously reported (22) using Total T4 ELISA (Biozol) and Mouse Pituitary Magnetic Bead Panel kits (MPTMAG-49K, MilliporeSigma) according to the manufacturer’s protocols with Victor plate reader devices (Perkin Elmer) and Luminex 200 (Luminex Corporation), respectively.

For the histology analysis, 4 μm–thick FFPE sections were stained with H&E using standard methods (22).

### Statistics.

The cAMP concentration (expressed as log_2_ fold change [log_2_FC] to WT) in basal condition and after TSH or hCG stimulation were tested with 2-way ANOVA, whereas relative cell membrane TSHR expression was tested with 1-way ANOVA. Both tests were corrected for multiple hypotheses by controlling the FDR using the 2-step Benjamini-Yekutieli method (72). The hCG-response assay was tested separately for basal and TSH-stimulated cAMP production (expressed as log_2_ FC to WT) using univariate ANOVA. Family-wise error rate was controlled using Dunnett’s method, considering WT hTSHR as the reference group in both conditions. In in vivo experiments, the statistical analysis was carried out using the 1-way ANOVA with Šidák multiple comparison, Brown-Forsythe and Welch ANOVA tests with Dunn’s multiple comparison, or Kruskal-Wallis tests with Dunn’s multiple comparison as described in figure legends, as appropriate. Regardless of the statistical method used, significance was considered when *P* < 0.05. All statistical methods were performed using GraphPad Prism 10 software (GraphPad Software).

### Study approval.

The Ethics Committee of the Hospital District of Southwest Finland approved this study (108/180/2010). The FinnGen project has been approved by the Ethical Review Board of the Hospital District of Helsinki and Uusimaa with the protocol no. HUS/990/2017. All FinnGen DNA samples and data evaluated in this study were pseudonymized. All animal experiments were authorized by the National Animal Experiment Board of Finland with license no. 35039. The patients and their family members were recruited to the study by their pediatric or adult endocrinologists. A written informed consent was obtained from all the study participants.

### Data availability.

Values for all data points in graphs are reported in the Supporting Data Values file.

## Author contributions

KM, MJ, KP, MPR, and JK conceptualized the study. KM, MJ, JK, and ML performed the sequencing and interpretation of the data. KM, RR, VL, JO, and VM performed and analyzed the murine data. MPR evaluated the FinnGen data. KM, MJ, PM, SL, MO, JJ, ML, FB, BS, KS, and AB recruited and characterized the clinical data. KM, MJ, LC, CL, MK, KP, RR, VL, JO, VM, and JK analyzed all the data. KM, LC, and JAM generated the TSHR constructs and participated to the in vitro tests. GK and PS analyzed the mutations using current TSHR structures and evaluated the whole manuscript. MJ, KM, and JK wrote the draft of the manuscript, and all authors contributed to the writing and editing of the final version. FinnGen provided data for the genetic database study.

## Supplementary Material

Supplemental data

Supporting data values

## Figures and Tables

**Table 1:**
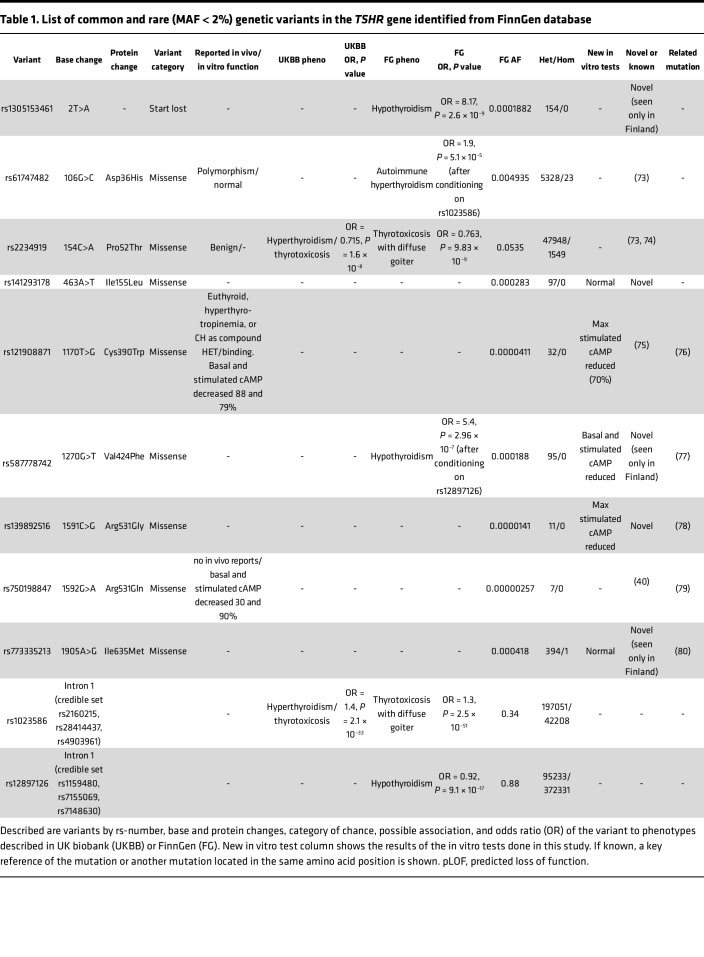
List of common and rare (MAF < 2%) genetic variants in the *TSHR* gene identified from FinnGen database

## Version 1 01/09/2024

In-Press Preview

## Version 2 02/22/2024

Electronic publication
