# Supplementary material for: Mechanisms of thyrotropin receptor–mediated phenotype variability deciphered by gene mutations and M453T-knockin model
Source: JCI Insight. 2024 Jan 9;9(4):e167092. doi: 10.1172/jci.insight.167092 (PMC11143923; doi:10.1172/jci.insight.167092)
Supplement: Supplemental data [file jciinsight-9-167092-s022.pdf]

Table S1: *Characteristics of the study participants.*

ID	TSHR/ other mutation	Sex	Age (yrs)	TSH 0.51 – 4.3 mU/L	FT4 11-20 pmol/L	FT3 2.6-6.3 pmol/L	uS-TSH/ n-TSH <40/<15mU/L	TPO_{Ab} <25IU/ml	$TSHR_{Ab}$ <1.9IU/L	Tygl *µg/L*	$Tygl_{Ab}$ <40IU/ml	HbA1c %	Cortisol nmol/L	ACTH ng/L	Thyroid size	Bone age*
67	*Arg519Cys*	*F*	*0.2*	*29*	*14.2*	-	*16*	*neg*	-	-	-	-	-	-	*n*	-
68	*Arg519Cys*	*F*	*0.6*	*48*	*24.7*	-	*17*	*neg*	-	-	-	-	-	-	*n*	-
74	*Ile640Val*	*F*	*24*	*0.015*	*17*	*5.5*	-	*neg*	*neg*	-	*neg*	*5.6*	*419*	*15*	*n*	-
75	*Ile640Val*	*M*	*2*	*0.021*	*18*	*7.2*	*<40*	*neg*	*neg*	-	*neg*	*5.5*	*318*	*21*	*n*	*0*
121	*Leu629Phe*	*M*	*0*	*<0.03*	*73.3*	-	*<40*	*neg*	*neg*	-	*neg*	-	*125*	-	*n*	*+/1.3*
Father of 121	*WT*	*M*	*50*	*1.54*	*17.5*	-	-	*neg*	-	-	*neg*	-	-	-	-	-
Mother of 121	*WT*	*F*	*45*	*3.39*	*13.0*	-	-	*neg*	*neg*	-	*neg*	-	-	-	-	-
Brother 1 of 121	*WT*	*M*	*20*	*1.53*	*14.2*	-	-	-	-	-	-	-	-	-	-	-
Brother 2 of 121	*WT*	*M*	*18*	*2.34*	*13.9*	-	-	-	-	-	-	-	-	-	-	-
137	*Ala519Cys*	*M*	*37*	*4.04*	*16.5*	-	-	*neg*	-	-	-	-	-	-	-	-
139	*WT/TG Asp2318Asn*	*F*	*44*	*norma l*	*normal*	-	-	*neg*	-	-	-	-	-	-	*goiter*	-
143	*Pro162Ala*	*M*	*7*	*22.1*	*12*	*6.3*	*25*	*neg*	-	-	-	-	-	-	-	-
144	*Pro162Ala*	*F*	*32*	*norma l*	*normal*	-	-	*neg*	-	-	*neg*	-	-	-	-	-
145	*Pro162Ala*	*M*	*41*	*norma l*	*normal*	-	-	*neg*	-	-	*neg*	-	-	-	-	-
229	*Asp633His*	*F*	*14*	*0.06*	*23.6*	*17.6*	-	*neg*	*neg*	-	-	-	-	-	*Right lobe: adenoma*	-
230	*Tyr601Asn*	*M*	*9*	*<0.03*	*31.5*	-	-	*neg*	*neg*	-	-	-	-	-	*Multinodu lar adenomas*	-
266	*Ile640Val*	*F*	*16*	*0.016*	*16*	*5.1*	*<40*	*neg*	*neg*	-	-	-	-	-	*normal*	-

ID	TSHR/ other mutation	Sex	Age (yrs)	TSH 0.51 – 4.3 mU/L	FT4 11-20 pmol/L	FT3 2.6-6.3 pmol/L	uS-TSH/ n-TSH <40/<15mU/L	TPO$_{Ab}$ <25IU/ml	TSHR$_{Ab}$ <1.9IU/L	Tygl µg/L	Tygl$_{Ab}$ <40IU/ml	HbA1c %	Cortisol nmol/L	ACTH ng/L	Thyroid size	Bone age*
Daughter of 266	*WT*	*F*	*2*	*1.9*	*16.1*	*3.9*	*<40*	*neg*	*-*	*23*	*neg*	-	-	-	-	-
Son of 266	*WT*	*M*	*4*	*2.4*	*16.7*	*6.2*	*<40*	*neg*	*-*	*17*	*neg*	-	-	-	-	-
268	*Ser237Asn*	*F*	*26*	*0.05*	*69.0*	*26.1*	*-*	*neg*	*neg*	*-*	*neg*	*-*	*-*	*-*	*-*	*-*
269	*Ser237Asn*	*F*	*40*	*0.02*	*74.5*	*13.7*	*-.*	*neg*	*neg*	*-*	*neg*	*-*	*-*	*-*	*-*	*-*
270	*Ser237Asn*	*M*	*13*	*0.005*	*35.4*	*11.7*	*<15*	*neg*	*neg*	*-*	*neg*	*4.61%*	*445*	*-*	*n, hypoecho and ↑ blood flow*	**+/3.0**
271	*Ser237Asn*	*F*	*10*	*0.01*	*29.5*	*14.1*	*<15*	*neg*	*neg*	*-*	*neg*	*-*	*-*	*-*	*normal*	+
272	*WT*	*F*	*?*	*0.9*	*-*	*-*	*<15*	*neg*	*-*	*-*	*-*	*-*	*-*	*-*	*-*	*-*
273	*Ser237Asn*	*F*	*14*	*0.01*	*37.3*	*17.6*	*-*	*neg*	*neg*	*-*	*neg*	*-*	*-*	*-*	*-*	*-*
274	*WT*	*M*	*14*	*1.54*	*-*	*-*	*<15*	*neg*	*-*	*-*	*-*	*-*	*-*	*-*	*-*	*-*
275	*WT*	*M*	*38*	*1.81*	*-*	*-*	*<15*	*-*	*-*	*-*	*-*	*-*	*-*	*-*	*-*	*-*
276	*WT*	*M*	*12*	*2.1*	*-*	*-*	*<15*	*neg*	*-*	*-*	*-*	*-*	*-*	*-*	*-*	*-*
279	*Ile640Val*	*F*	*5*	*0.06*	*23.0*	*7.0*	*<40*	*neg*	*neg*	*11.2*	*neg*	*-*	*342*	*21*	*normal/ 2 small nodules (7x7x3mm)*	*-*
280	*Ile640Val*	*M*	*41*	*0.04*	*17.0*	*5.2*	*-*	*neg*	*neg*	*0.7*	*-*	*-*	*388*	*-*	*normal*	*-*
283	*WT*	*F*	*45*	*2.05*	*17.0*	*-*	*-*	*neg*	*neg*	*34.2*	*neg*	*-*	*-*	*-*	*-*	*-*
284	*WT*	*F*	*19*	*3.91*	*-*	*-*	*-*	*neg*	*-*	*11*	*neg*	*-*	*-*	*-*	*-*	*-*
285	*Ile640Val*	*F*	*12*	*0.85*	*19*	*-*	*3.4*	*neg*	*neg*	*11*	*neg*	*-*	*-*	*-*	*-*	*-*
286	*WT*	*M*	*10*	*3.91*	*18*	*-*	*8.7*	*neg*	*neg*	*31.4*	*neg*	*4.8*	*-*	*-*	*-*	*-*
291	*WT*	*M*	*1.7*	*1.9*	*14*	*-*	*<40*	*-*	*-*	*-*	*-*	*-*	*-*	*-*	*-*	*-*
292	*Ala485Val/ TSHR Val233Met*	*M*	*1.1*	*0.01*	*20*	*9.0*	*1.78*	*neg*	*neg*	*-*	*neg*	*-*	*-*	*-*	*normal*	*-*

ID	TSHR/ other mutation	Sex	Age (yrs)	TSH 0.51 – 4.3 mU/L	FT4 11-20 pmol/L	FT3 2.6-6.3 pmol/L	uS-TSH/ n-TSH <40/<15mU/L	TPO$_{Ab}$ <25IU/ml	TSHR$_{Ab}$ <1.9IU/L	Tygl *µg/L*	Tygl$_{Ab}$ <40IU/ml	HbA1c %	Cortisol nmol/L	ACTH ng/L	Thyroid size	Bone age*
293	*Ala485Val/ TSHR Val233Met*	*M*	*4.8*	*0.01*	*57*	*-*	*0.03*	*neg*	*neg*	*-*	*neg*	*4.7*	*280*	*7*	*normal*	*-*
294, mother of 291-3	*WT/ TSHR Val233Met*	*F*	*27*	*5.0*	*17*	*-*	*-*	*neg*	*-*	*24.5*	*neg*	*-*	*-*	*-*	*-*	*-*
295, father of 291-3	*Ala485Val/ WT*	*M*	*32*	*0.01*	*17*	*7.7*	*-*	*neg*	*-*	*24.7*	*neg*	*-*	*-*	*-*	*normal/1 cyst and 1 small nodule*	*-*

*Characteristics of the study participants including number (ID), TSHR or other gene mutation, Age at diagnosis or at enrollment, thyroid function test results (TSH (Ref. 0.1-17 mU/L in newborns, 0.5-4.0 mU/L in adults), free T4 (FT4, Ref. 10 – 36 pmol/l in 1 – 3 day-old newborns and 9.0 – 19 pmol/l in adults) or free T3 (FT3) concentrations (Ref. 3.5 - 7.5 , pmol/L), uS-TSH= umbilical cord blood serum TSH (used in Finland for CH screening, Reference values for uS-TSH < 40mU/L), n-TSH, neonatal TSH at screening (used in Poland, measured at 3 days of age from heel prick blood sample. Screening limit for n-TSH was < 15 mU/L. Thyroid peroxidase (TPO), TSHR, or Thyroglobulin, (Tygl) antibodies (Ab) neg, indicates negative antibody test result, HbA1c, glycated hemoglobin A1c, ACTH, adrenocorticotrophic hormone. Thyroid size was evaluated with ultrasound, n = normal thyroid size and location. *Bone age,* years ahead (+/y) or behind (-/y) *calendar age, estimated from hand X-ray using the Tanner-Whitehouse skeletal maturity method (1).*

Table S2: *Thyroid-specific rare exome variants in study subjects with nonautoimmune hyper- or hypothyroidism.*

ID	Gene	GeneBank	Base change	Protein change	gnomAD MAF	Chr location (GRCh38.p13)	Novel or reference	Classification*
67	*TSHR*	*NM_000369.2*	*c.1555C>T*	*R519C*	*0.00001769*	*Chr14: 81143613*	*(2)*	*A*
68	*TSHR*	*NM_000369.2*	*c.1555C>T*	*R519C*	*0.00001769*	*Chr14: 81143613*	*(2)*	*A*
67	*TG*	*NM_003235.4*	*c.6952G>A*	*D2318N*	*0.0004030*	*Chr8: 133022066*	-	*VUS*
68	*TG*	*NM_003235.4*	*c.6952G>A*	*D2318N*	*0.0004030*	*Chr8: 133022066*	-	*VUS*
74	*TSHR*	*NM_000369.2*	*c.1918A>G*	*I640V*	-	*Chr14: 81 143 976*	*Novel*	*A*
75	*TSHR*	*NM_000369.2*	*c.1918A>G*	*I640V*	-	*Chr14: 81 143 976*	*Novel*	*A*
121	*TSHR*	*NM_000369.2*	*c.1887G>C*	*L629F*	*0.000003977*	*Chr14: 81 143 945*	*(3)*	*A*
143	*TSHR*	*NM_000369.2*	*c.484C>G*	*P162A*	*0.0001379*	*Chr14:81 092 547*	*(4)*	*A*
229	*TSHR*	*NM_000369.2*	*c.1897G>C*	*D633H*	*0.000003977*	*Chr14: 81 143 955*	*(5)*	*A*
230	*TSHR*	*NM_000369.2*	*c.1801T>A*	*Y601N*	-	*Chr14: 81 143 859*	*(6)*	*A*
230	*PAX8*	*NM_003466.4*	*c.985C>T*	*F329L*	*0.01587*	*Chr2:113 235 496*	*(7)*	*B*
266	*TSHR*	*NM_000369.2*	*1918A>G*	*I640V*	-	*Chr14: 81 143 976*	*Novel*	*A*
268	*TSHR*	*NM_000369.2*	*c.710G>A*	*S237N*	-	*Chr14: 81 139 696*	*Novel*	*A*
292	*TSHR*	*NM_000369.2*	*c.1454C>T*	*A485V*	-	*Chr14: 81 143 512*	*(8)*	*A*
293	*TSHR*	*NM_000369.2*	*c.697G>A*	*V233M*	*0.00003536*	*Chr14:81 139 683*	*(9)*	*B*

**Classification: ID = number of the participant, A = pathogenic mutation (based on segregation, literature, and in vitro experiments), B = benign variant, VUS = variant of unknown significance, gnomAD MAF = minor allele frequency in The Genome Aggregation Consortium database.*

Table S3: *List of TSHR primers used for Sanger-sequencing.*

**Target site in *TSHR***	**Forward primer**	**Reverse primer**	T_a
p.Val233Met (c.697G>A)	*5'-AGTCCCCAAACTCTAGTCCCC-3'*	*5'-GGTAAGAAAGGTCAGCCCGTG-3'*	*65 °C*
p.Ser237Asn (c.710G>A)	*5'-AGTCCCCAAACTCTAGTCCCC-3'*	*5'-GGTAAGAAAGGTCAGCCCGTG-3'*	*65 °C*
p.Ala485Val (c.1454C>T)	*5'-TCCGATGAGTTCAACCCGTG-3'*	*5'-GGTGATGACCGTCAGCGTAT-3'*	*64°C*
p.Leu629Phe (c.1887G>C)	*5'-GTCAGTATCTGCCTGCCCAT-3'*	*5'-CTGAGCCTGGCGTTTACAGA-3'*	*64 °C*
p.Tyr601Asn (c.1801T>A)	*5'- CCGAGACCCCTCTTGCTCTG-3'*	*5'-CCAGCAAGATTTTGGAGTTGCT-3'*	*63 °C*
p.Asp633His (c.1897G>C)	*5'- CCGAGACCCCTCTTGCTCTG-3'*	*5'-CCAGCAAGATTTTGGAGTTGCT-3'*	*63 °C*
p.Ile640Val (c.1918A>G)	*5'-GTCAGTATCTGCCTGCCCAT-3'*	*5'-CTGAGCCTGGCGTTTACAGA-3'*	*64 °C*

T_a, annealing temperature of the primer pairs.

Table S4: *List of primers used for quantitative RT-PCR.*

Gene	Forward primer	Reverse primer
Nis	*5'-GGGATGCACCAATGCCTCTG-3'*	*5'-GTAGCTGATGAGAGCACCACA-3'*
Ppia	*5'-CATCCTAAAGCATACAGGTCCTG-3'*	*5'-TCCATGGCTTCCACAATGTT-3'*
Tpo	*5'-CAAAGGCTGGAACCCTAATTTCT-3'*	*5'-AACTTGAATGAGGTGCCTTGTCA-3'*

Annealing temperature for the PCR was 62.5°C for all the primers.

Table S5: *List of antibodies*

Primary antibodies

FACS

- Rabbit monoclonal HA-tag IgG (C29F4); Cell Signaling Technology (37245)

Immunohistochemistry

- Rat monoclonal Ki-67 (SolA15); eBioscience™ (11-5698-82)
- Rabbit polyclonal Anti-Thyroid Peroxidase (TPO); Abcam (ab203057)

Secondary antibodies

FACS

- Donkey anti-Rabbit IgG (H+L) Highly Cross-Adsorbed Secondary Antibody, Alexa Fluor™ 488; Invitrogen (A-21206)
- Donkey anti-Rabbit IgG (H+L) Highly Cross-Adsorbed Secondary Antibody, Alexa Fluor™ 647; Invitrogen (A-31573)

Immunohistochemistry

- Rat on Mouse HRP-Polymer Kit; Biocare (RT517)
- Goat anti-rabbit HRP; WellMed (DPVR110HRP)

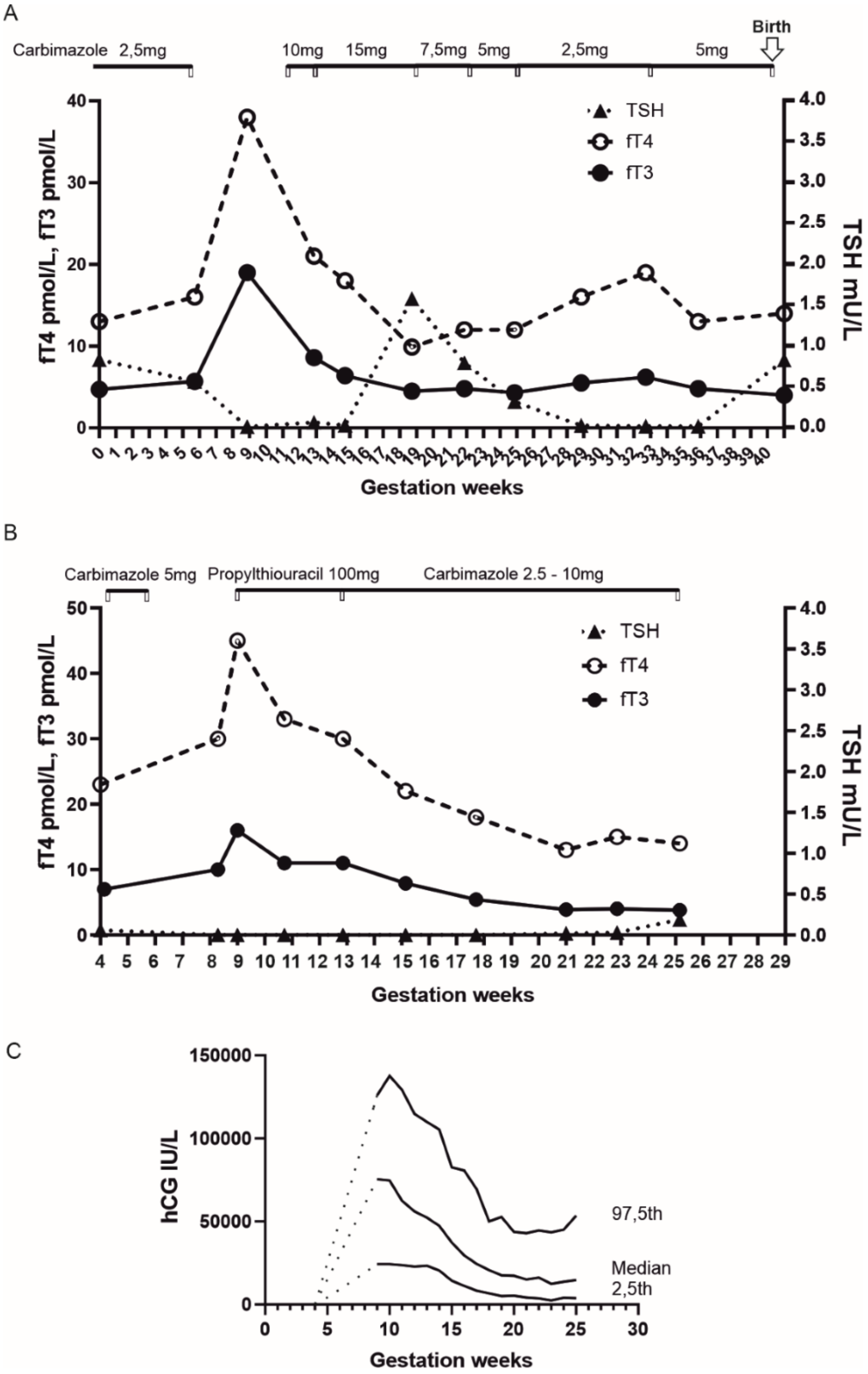
A
Carbimazole 2,5mg
10mg 15mg 7,5mg 5mg 2,5mg 5mg
Birth
TSH
fT4
fT3
fT4 pmol/L, fT3 pmol/L
TSH mU/L
Gestation weeks
B
Carbimazole 5mg
Propylthiouracil 100mg
Carbimazole 2.5 - 10mg
TSH
fT4
fT3
fT4 pmol/L, fT3 pmol/L
TSH mU/L
Gestation weeks
C
hCG IU/L
97,5th
Median
2,5th
Gestation weeks

Figure S1. *Thyroid function tests (TSH, T4v and T3v concentrations) and a dose of antithyroid medication during pregnancy of the patients* **A)** *#266 and* **B)** *#279 with TSHR CAM Ile640Val mutation.* **C)** *Graph of gestational age specific reference ranges for total human chorionic gonadotropin (hCG) levels during pregnancy modified from Korevaar et al. (10) showing a peak in the 9th and 10th week of gestation.*

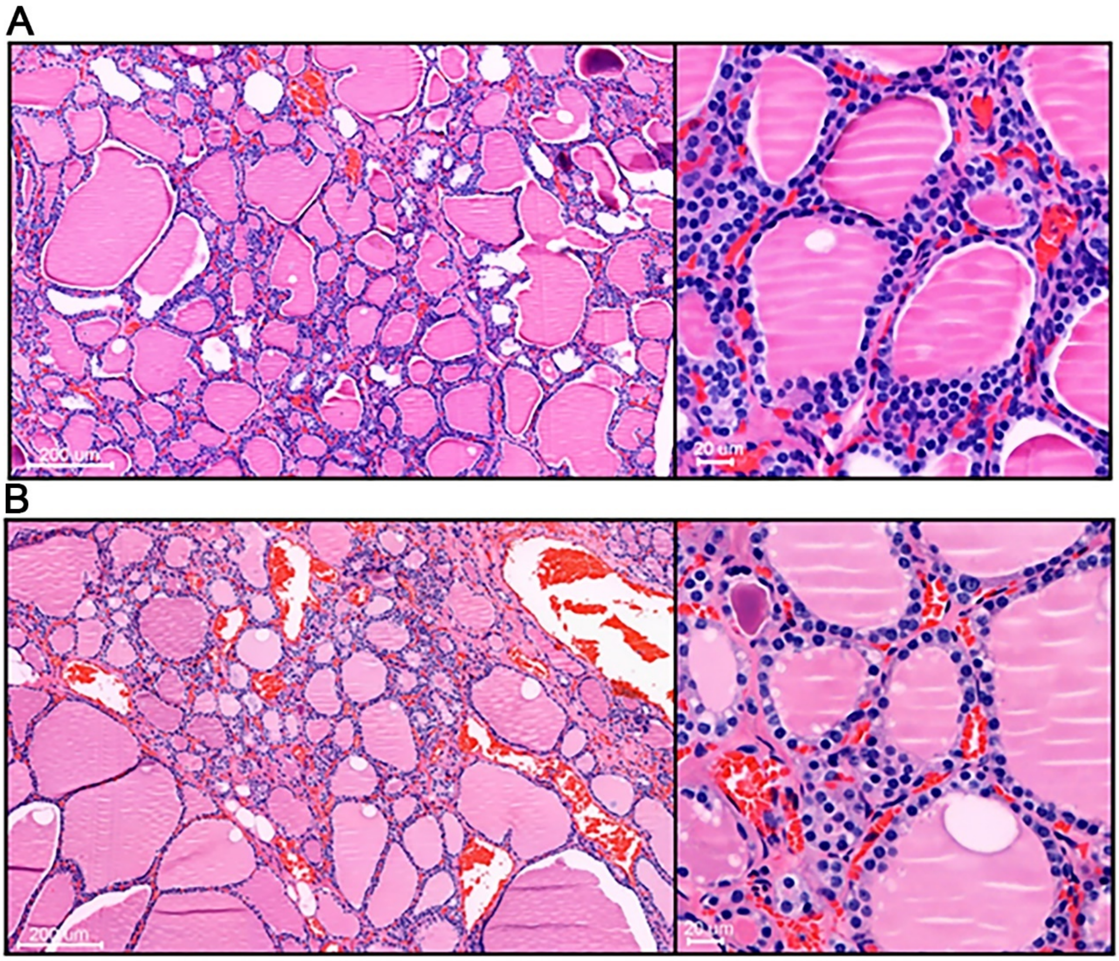

Figure S2. *Hematoxylin-eosin-stained thyroid sections of the individuals* **A)** *#266 and* **B)** *#280 with TSHR Ile640Val mutation.*

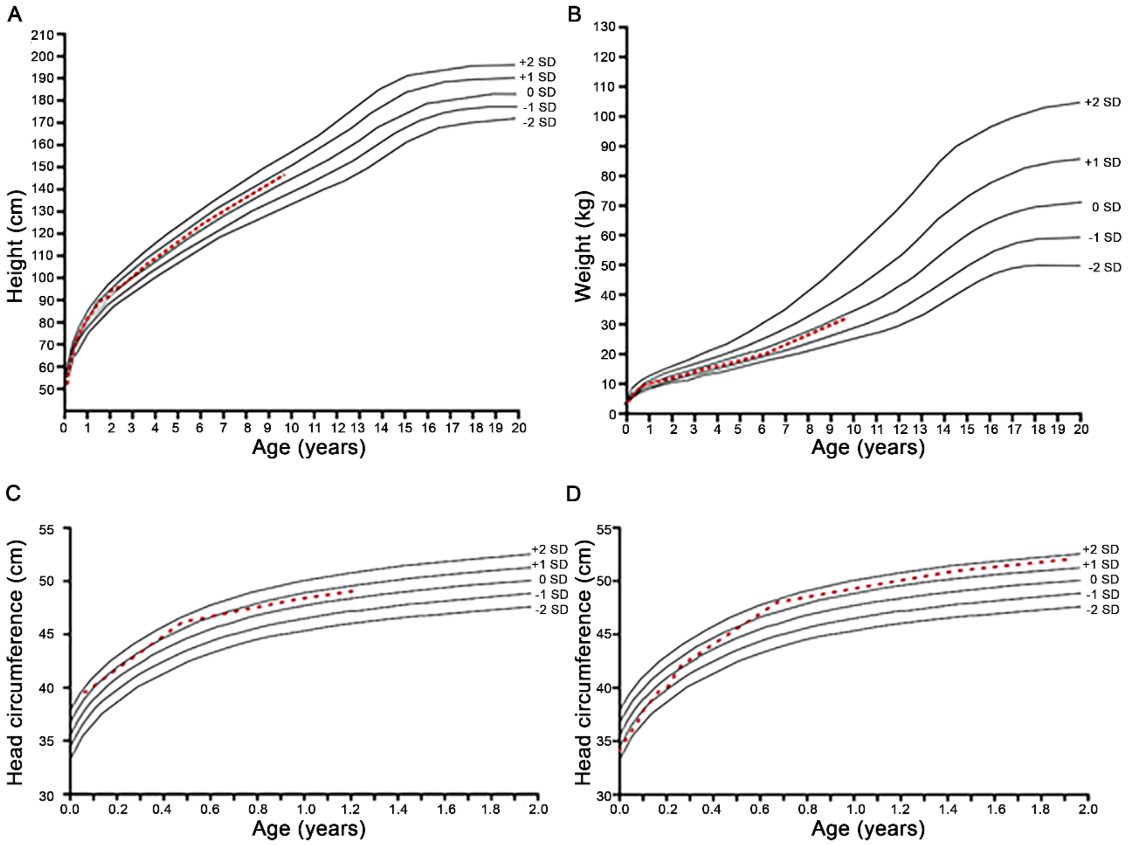

Figure S3. *Additional growth-, head growth- and weight curves of the children with TSHR CAM mutations. Patient (#75) with TSHR Ile640Val mutation and subclinical hyperthyroidism showing normal* **A)** *weight gain,* **B)** *linear growth, and* **C)** *head growth.* **D)** *Head growth curve of the patient (#121) with TSHR L629F mutation during the first two years of his life.*

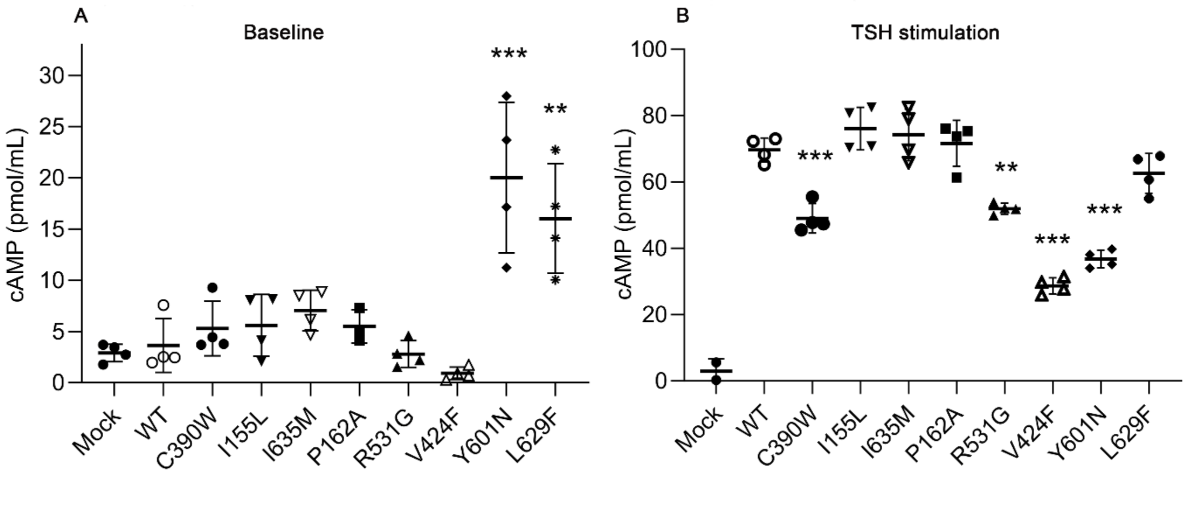

Figure S4. *Panel A basal and Panel B TSH-stimulated (10mU/ml, lower panel) cAMP secretion of WT and TSHR mutants detected in FinnGen database. Two previously described constitutively active TSHR mutations Y601N and L629F were used as positive controls. Statistical comparisons were calculated using one-way ANOVA with Bonferroni's multiple comparisons test. **p< 0.01; ***p< 0.001.*

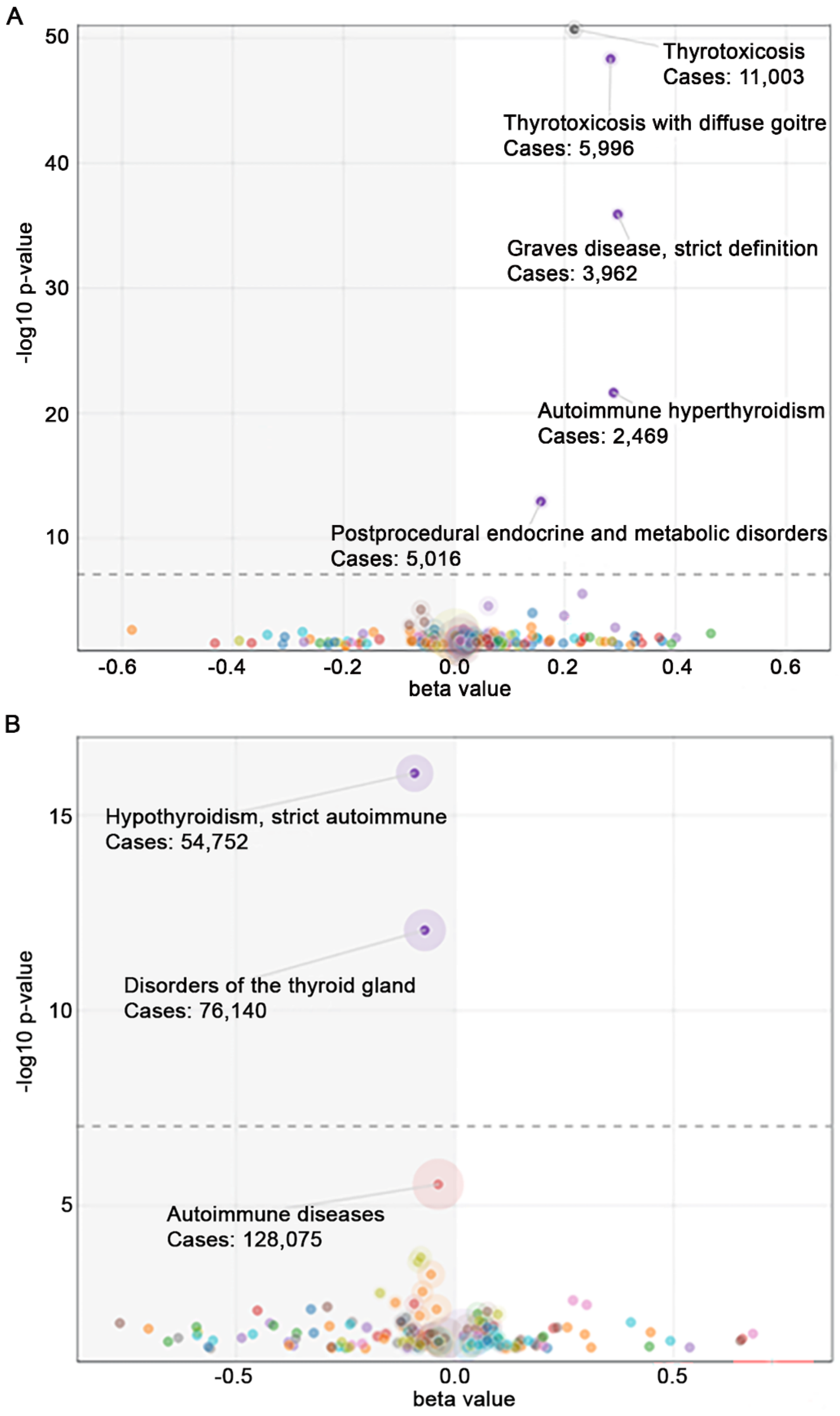

Figure S5. *An association of the TSHR variants to disease phenotypes in FinnGen database. Panel A variant rs1023586 (new lead SNP for rs179252 Thyrotoxicosis, TSHR intron 1), and Panel B rs12897126 (TSHR hypothyroidism lead) with the associated risk to hypothyroidism.*

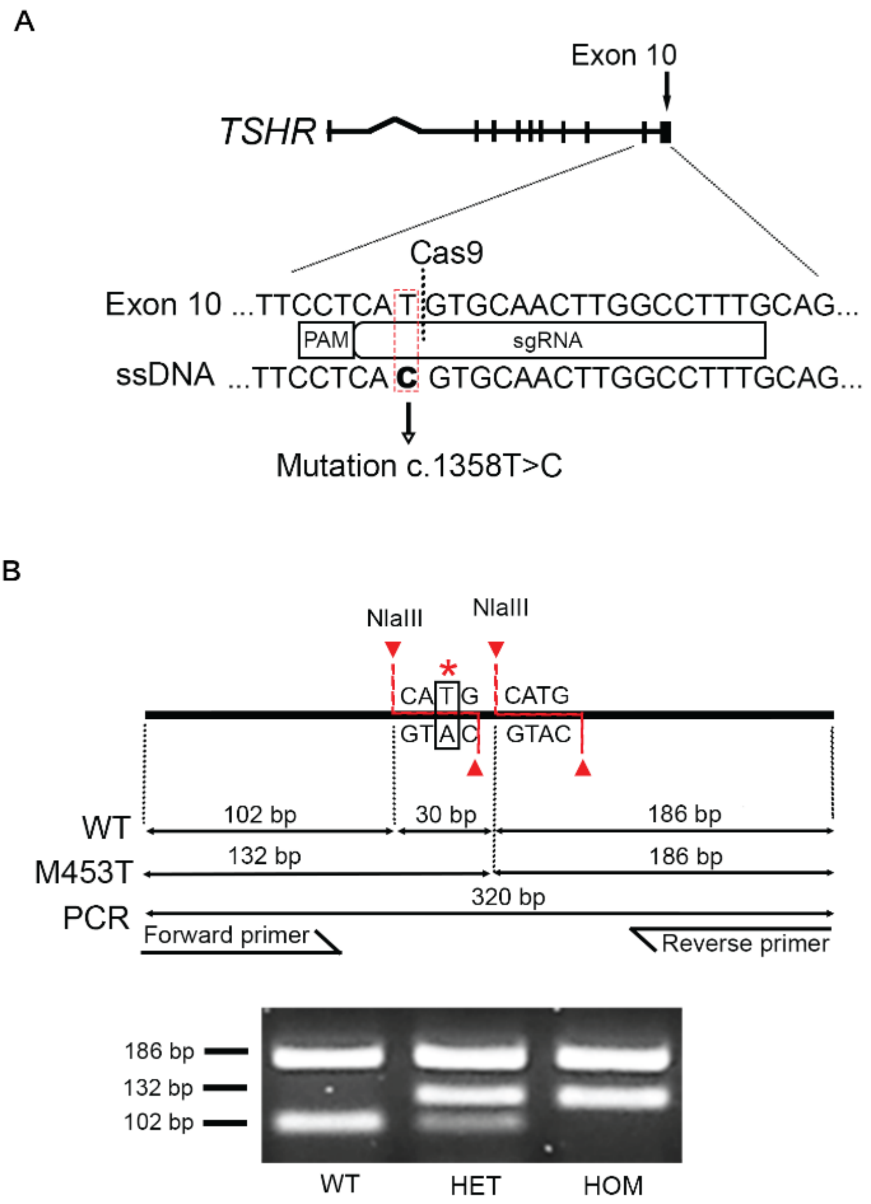

Figure S6. *Generation and genotyping of the TSHR M453T knock-in mice using CRISPR/Cas9 and homology-directed repair technique.* **A)** *Genomic location and designed guide RNA (gRNA) construct.* **B)** *Middle panel shows, PCR primer locations, expected sizes and location of NlaIII restriction site altered by the TSHR c.1358 T>C mutation. Below is an example of agarose gel electrophoresis with different WT, HET, and HOM genotypes after digestion of the PCR product with NlaIII restriction enzyme.*

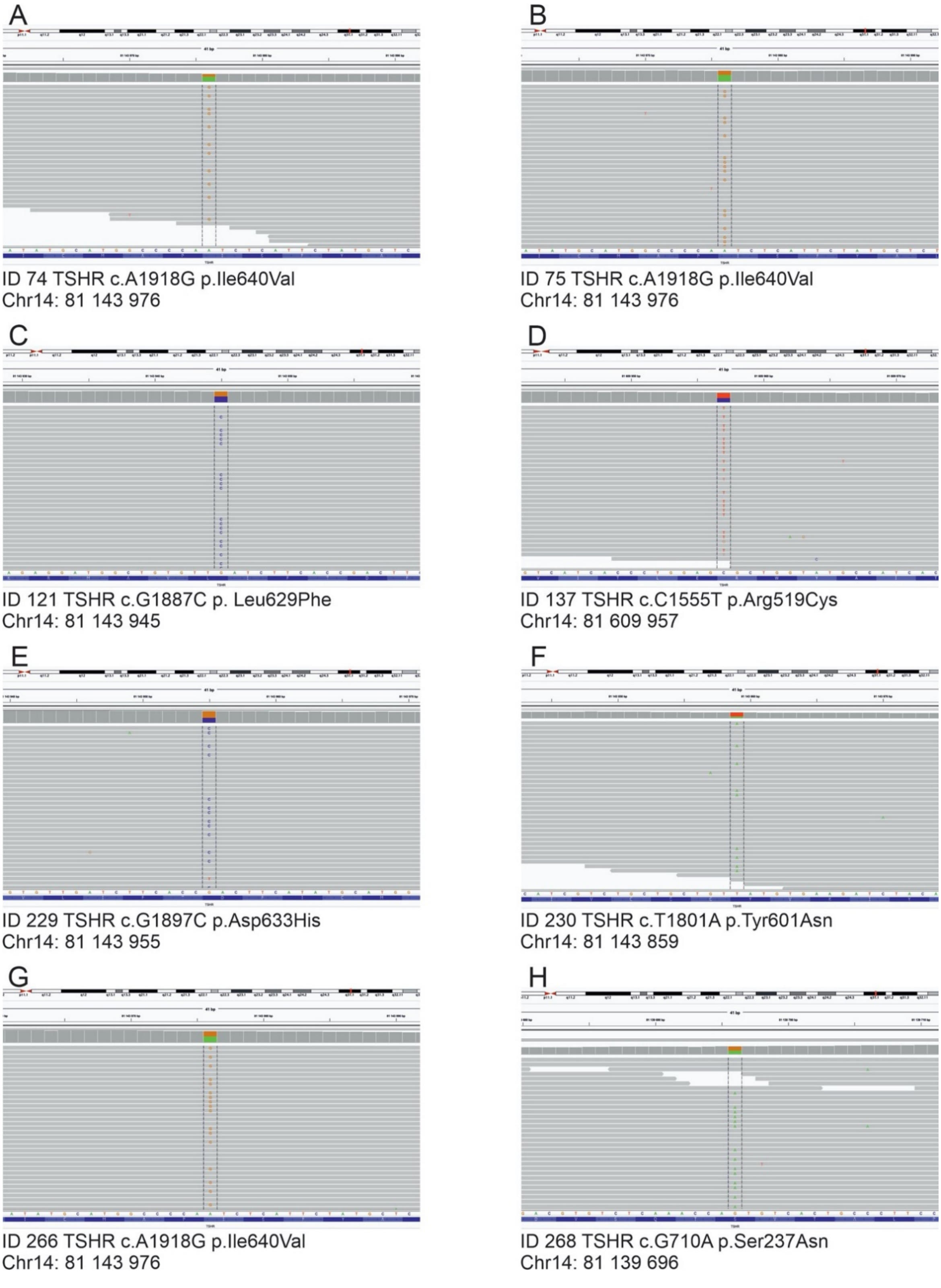

Figure S7. *All TSHR mutations detected in this study by next-generation-sequencing were visualized using the Integrative Genomics Viewer (11). Example IGV pictures of* **A)** *ID74 and* **B)** *ID74 with TSHR c.1918A>G,* **C)** *D121, TSHR c.1887G>C, D) ID137, TSHR c.1555 C>T,* **E)** *ID229, TSHR c.1897G>C,* **F)** *ID 230, TSHR c.1801T>A,* **G)** *ID266, TSHR c.1918 A>G and* **H)** *ID268 with TSHR c.710G>A mutation.*

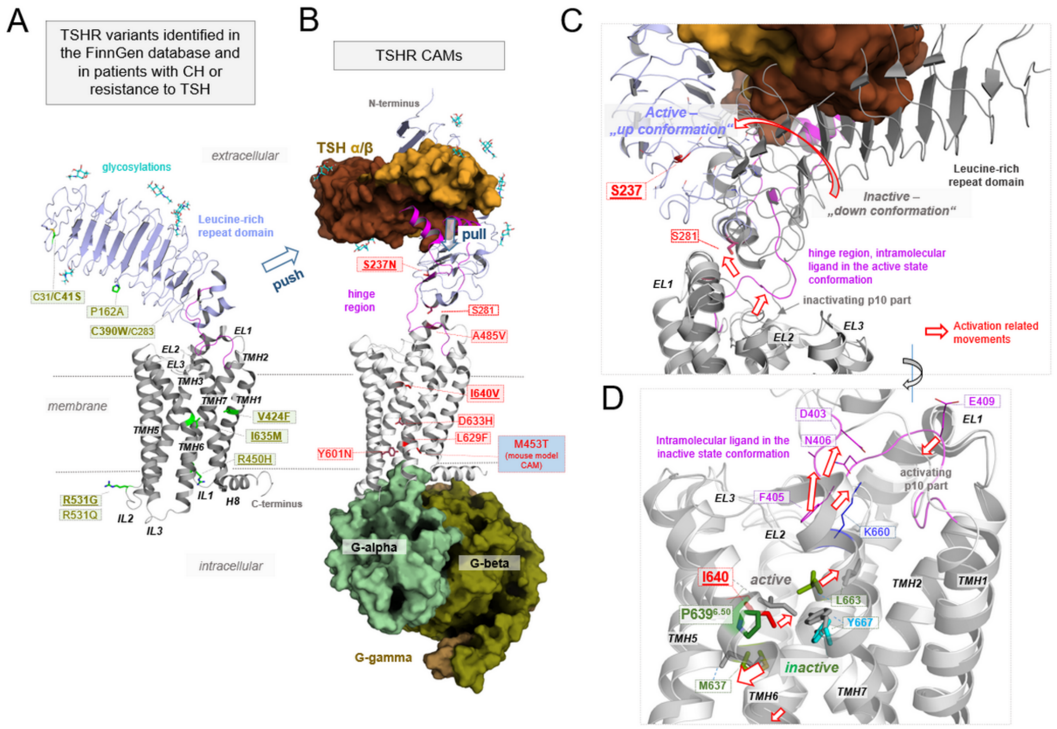

Figure S8. Structural and molecular insights into TSHR variants identified and investigated in this study. *Recently determined TSHR WT structures in different activity state conformations were used to highlight the impact of the described TSHR variants on TSHR structure-function relationships.* **A)** *The inactive state TSHR WT structure (PDB ID 7t9m (12)) with TSHR variants identified in the FinnGen database and in patients with CH or resistance to TSH. Underlined are newly identified variants.* **B)** *The active WT TSHR state structure (PDB ID 7t9i (13)) is highlighted with the CAMs identified and shown in this study. TSH and the G-protein molecules are presented as surfaces with different colors. The receptor is visualized by a backbone cartoon. The hormone binding induced push-movement of the extracellular LRRD/hinge-TSH complex is indicated, as well as the pull-mechanism via the TSH-hinge interaction. In* **C)** *and* **D)** *both complexes are in superimposition for comparison at specific structural regions important to explain molecular backgrounds of receptor activation and the associated potential models of constitutive receptor activation. TMH – transmembrane helix, EL – extracellular loop, IL – intracellular loop.*

Supplementary Materials and Methods

Quantitative real-time PCR

TPO and NIS gene expression was studied for female wildtype and homozygous TSHR M453T mice on sufficient- and high-iodine diet using quantitative real-time PCR (RT-PCR). Total RNA was extracted from thyroid tissue using NucleoSpin RNA isolation kit (MACHEREY-NAGEL GmbH & Co.). For cDNA synthesis, 300ng RNA was treated with deoxyribonuclease I (InvitrogenTM) and reverse transcribed using SensiFAST cDNA Synthesis Kit (meridian BIOSCIENCETM). A cDNA dilution of 1:10 was used for RT-PCR (BIO-RAD- CFX96 Real-Time System), performed using Power SYBR® Green PCR Master Mix (Applied Biosystems, Inc.). All the experiments were conducted according to the manufacturer's protocol. The samples were run in duplicates. PPIA was used as an endogenous control for normalization. The primer sequences used for TPO, NIS and PPIA **(Supplemental Table S4)** *have been previously published (14). The relative fold change was calculated using 2^-ΔΔCt method. Statistical analysis was performed using GraphPad Prism 10.*

Ethics statement and materials and methods related to FinnGen analysis

Study subjects in FinnGen provided informed consent for biobank research, based on the Finnish Biobank Act. Alternatively, separate research cohorts, collected prior the Finnish Biobank Act came into effect (in September 2013) and start of FinnGen (August 2017), were collected based on study-specific consents and later transferred to the Finnish biobanks after approval by Fimea (Finnish Medicines Agency), the National Supervisory Authority for Welfare and Health. Recruitment protocols followed the biobank protocols approved by Fimea. The Coordinating Ethics Committee of the Hospital District of Helsinki and Uusimaa (HUS) statement number for the FinnGen study is Nr HUS/990/2017.

The FinnGen study is approved by Finnish Institute for Health and Welfare (permit numbers: THL/2031/6.02.00/2017, THL/1101/5.05.00/2017, THL/341/6.02.00/2018, THL/2222/6.02.00/2018, THL/283/6.02.00/2019, THL/1721/5.05.00/2019 and THL/1524/5.05.00/2020), Digital and population data service agency (permit numbers: VRK43431/2017-3, VRK/6909/2018-3, VRK/4415/2019-3), the Social Insurance Institution (permit numbers: KELA 58/522/2017, KELA 131/522/2018, KELA 70/522/2019, KELA

98/522/2019, KELA 134/522/2019, KELA 138/522/2019, KELA 2/522/2020, KELA 16/522/2020), Findata permit numbers THL/2364/14.02/2020, THL/4055/14.06.00/2020, THL/3433/14.06.00/2020, THL/4432/14.06/2020, THL/5189/14.06/2020, THL/5894/14.06.00/2020, THL/6619/14.06.00/2020, THL/209/14.06.00/2021, THL/688/14.06.00/2021, THL/1284/14.06.00/2021, THL/1965/14.06.00/2021, THL/5546/14.02.00/2020, THL/2658/14.06.00/2021, THL/4235/14.06.00/2021, Statistics Finland (permit numbers: TK-53-1041-17 and TK/143/07.03.00/2020 (earlier TK-53-90-20) TK/1735/07.03.00/2021, TK/3112/07.03.00/2021) and Finnish Registry for Kidney Diseases permission/extract from the meeting minutes on 4th July 2019.

The Biobank Access Decisions for FinnGen samples and data utilized in FinnGen Data Freeze 11 include: THL Biobank BB2017_55, BB2017_111, BB2018_19, BB_2018_34, BB_2018_67, BB2018_71, BB2019_7, BB2019_8, BB2019_26, BB2020_1, BB2021_65, Finnish Red Cross Blood Service Biobank 7.12.2017, Helsinki Biobank HUS/359/2017, HUS/248/2020, HUS/430/2021 §28, §29, HUS/150/2022 §12, §13, §14, §15, §16, §17, §18, §23, §58 and §59, Auria Biobank AB17-5154 and amendment #1 (August 17 2020) and amendments BB_2021-0140, BB_2021-0156 (August 26 2021, Feb 2 2022), BB_2021-0169, BB_2021-0179, BB_2021-0161, AB20-5926 and amendment #1 (April 23 2020) and it´s modification (Sep 22 2021), BB_2022-0262, BB_2022-0256, Biobank Borealis of Northern Finland_2017_1013, 2021_5010, 2021_5018, 2021_5015, 2021_5015 Amendment, 2021_5023, 2021_5023 Amendment, 2021_5017, 2022_6001, 2022_6006 Amendment, BB22-0067, 2022_0262, Biobank of Eastern Finland 1186/2018 and amendment 22§/2020, 53§/2021, 13§/2022, 14§/2022, 15§/2022, 27§/2022, 28§/2022, 29§/2022, 33§/2022, 35§/2022, 36§/2022, 37§/2022, 39§/2022, 7§/2023, Finnish Clinical Biobank Tampere MH0004 and amendments (21.02.2020 & 06.10.2020), 8§/2021, 9§/2021, §9/2022, §10/2022, §12/2022, 13§/2022, §20/2022, §21/2022, §22/2022, §23/2022, 28§/2022, 29§/2022, 30§/2022, 31§/2022, 32§/2022, 38§/2022, 40§/2022, 42§/2022, 1§/2023, Central Finland Biobank 1-2017, BB_2021-0161, BB_2021-0169, BB_2021-0179, BB_2021-0170, BB_2022-0256, and Terveystalo Biobank STB 2018001 and amendment 25th Aug 2020, Finnish Hematological Registry and Clinical Biobank decision 18th June 2021, Arctic biobank P0844: ARC_2021_1001.

FinnGen consortium participants:

Aarno Palotie, Mark Daly, Bridget Riley-Gills, Howard Jacob, Dirk Paul, Athena Matakidou, Adam Platt, Heiko Runz, Sally John, George Okafo, Nathan Lawless, Heli Salminen-Mankonen, Robert Plenge, Joseph Maranville, Mark McCarthy, Margaret G Ehm, Kirsi Auro, Simonne Longerich, Caroline Fox, Anders Mälarstig, Katherine Klinger, Clement Chatelain, Matthias Gossel, Karol Estrada, Robert Graham, Robert Yang, Chris O Donnell, Tomi P Mäkelä, Jaakko Kaprio, Petri Virolainen, Antti Hakanen, Terhi Kilpi, Markus Perola, Jukka Partanen, Anne Pitkäranta, Taneli Raivio, Raisa Serpi, Tarja Laitinen, Veli-Matti Kosma, Jari Laukkanen, Marco Hautalahti, Outi Tuovila, Raimo Pakkanen, Jeffrey Waring, Bridget Riley-Gillis, Fedik Rahimov, Ioanna Tachmazidou, Chia-Yen Chen, Zhihao Ding, Marc Jung, Shameek Biswas, Rion Pendergrass, David Pulford, Neha Raghavan, Adriana Huertas-Vazquez, Jae-Hoon Sul, Xinli Hu, Åsa Hedman, Manuel Rivas, Dawn Waterworth, Nicole Renaud, Ma En Obeidat, Samuli Ripatti, Johanna Schleutker, Mikko Arvas, Olli Carpén, Reetta Hinttala, Arto Mannermaa, Katriina Aalto-Setälä, Mika Kähönen, Johanna Mäkelä, Reetta Kälviäinen, Valtteri Julkunen, Hilkka Soininen, Anne Remes, Mikko Hiltunen, Jukka Peltola, Minna Raivio, Pentti Tienari, Juha Rinne, Roosa Kallionpää, Juulia Partanen, Ali Abbasi, Adam Ziemann, Nizar Smaoui, Anne Lehtonen, Susan Eaton, Sanni Lahdenperä, Natalie Bowers, Edmond Teng, Fanli Xu, Laura Addis, John Eicher, Qingqin S Li, Karen He, Ekaterina Khramtsova, Martti Färkkilä, Jukka Koskela, Sampsa Pikkarainen, Airi Jussila, Katri Kaukinen, Timo Blomster, Mikko Kiviniemi, Markku Voutilainen, Tim Lu, Linda McCarthy, Amy Hart, Meijian Guan, Jason Miller, Kirsi Kalpala, Melissa Miller, Kari Eklund, Antti Palomäki, Pia Isomäki, Laura Pirilä, Oili Kaipiainen-Seppänen, Johanna Huhtakangas, Nina Mars, Apinya Lertratanakul, Marla Hochfeld, Jorge Esparza Gordillo, Fabiana Farias, Nan Bing, Margit Pelkonen, Paula Kauppi, Hannu Kankaanranta, Terttu Harju, Riitta Lahesmaa, Glenda Lassi, Hubert Chen, Joanna Betts, Rajashree Mishra, Majd Mouded, Debby Ngo, Teemu Niiranen, Felix Vaura, Veikko Salomaa, Kaj Metsärinne, Jenni Aittokallio, Jussi Hernesniemi, Daniel Gordin, Juha Sinisalo, Marja-Riitta Taskinen, Tiinamaija Tuomi, Timo Hiltunen, Amanda Elliott, Mary Pat Reeve, Sanni Ruotsalainen, Benjamin Challis, Audrey Chu, Dermot Reilly, Mike Mendelson, Jaakko Parkkinen, Tuomo Meretoja, Heikki Joensuu, Johanna Mattson, Eveliina Salminen, Annika Auranen, Peeter Karihtala, Päivi Auvinen, Klaus Elenius, Esa Pitkänen, Relja Popovic, Jennifer Schutzman, Diptee Kulkarni, Alessandro

Porello, Andrey Loboda, Heli Lehtonen, Stefan McDonough, Sauli Vuoti, Kai Kaarniranta, Joni A Turunen, Terhi Ollila, Hannu Uusitalo, Juha Karjalainen, Mengzhen Liu, Stephanie Loomis, Erich Strauss, Hao Chen, Kaisa Tasanen, Laura Huilaja, Katariina Hannula-Jouppi, Teea Salmi, Sirkku Peltonen, Leena Koulu, David Choy, Ying Wu, Pirkko Pussinen, Aino Salminen, Tuula Salo, David Rice, Pekka Nieminen, Ulla Palotie, Maria Siponen, Liisa Suominen, Päivi Mäntylä, Ulvi Gursoy, Vuokko Anttonen, Kirsi Sipilä, Hannele Laivuori, Venla Kurra, Laura Kotaniemi-Talonen, Oskari Heikinheimo, Ilkka Kalliala, Lauri Aaltonen, Varpu Jokimaa, Marja Vääräsmäki, Laure Morin-Papunen, Maarit Niinimäki, Terhi Piltonen, Katja Kivinen, Elisabeth Widen, Taru Tukiainen, Niko Välimäki, Eija Laakkonen, Heidi Silven, Riikka Arffman, Susanna Savukoski, Triin Laisk, Natalia Pujol, Janet Kumar, Iiris Hovatta, Erkki Isometsä, Hanna Ollila, Jaana Suvisaari, Thomas Damm Als, Antti Mäkitie, Argyro Bizaki-Vallaskangas, Sanna Toppila-Salmi, Tytti Willberg, Elmo Saarentaus, Antti Aarnisalo, Elisa Rahikkala, Kristiina Aittomäki, Fredrik Åberg, Mitja Kurki, Aki Havulinna, Juha Mehtonen, Priit Palta, Shabbeer Hassan, Pietro Della Briotta Parolo, Wei Zhou, Mutaamba Maasha, Susanna Lemmelä, Aoxing Liu, Arto Lehisto, Andrea Ganna, Vincent Llorens, Henrike Heyne, Joel Rämö, Rodos Rodosthenous, Satu Strausz, Tuula Palotie, Kimmo Palin, Javier Garcia-Tabuenca, Harri Siirtola, Tuomo Kiiskinen, Jiwoo Lee, Kristin Tsuo, Kati Kristiansson, Kati Hyvärinen, Jarmo Ritari, Katri Pylkäs, Minna Karjalainen, Tuomo Mantere, Eeva Kangasniemi, Sami Heikkinen, Nina Pitkänen, Samuel Lessard, Clément Chatelain, Perttu Terho, Tiina Wahlfors, Eero Punkka, Sanna Siltanen, Teijo Kuopio, Anu Jalanko, Huei-Yi Shen, Risto Kajanne, Mervi Aavikko, Henna Palin, Malla-Maria Linna, Masahiro Kanai, Zhili Zheng, L Elisa Lahtela, Mari Kaunisto, Elina Kilpeläinen, Timo P Sipilä, Oluwaseun Alexander Dada, Awaisa Ghazal, Anastasia Kytölä, Rigbe Weldatsadik, Kati Donner, Anu Loukola, Päivi Laiho, Tuuli Sistonen, Essi Kaiharju, Markku Laukkanen, Elina Järvensivu, Sini Lähteenmäki, Lotta Männikkö, Regis Wong, Auli Toivola, Minna Brunfeldt, Hannele Mattsson, Sami Koskelainen, Tero Hiekkalinna, Teemu Paajanen, Kalle Pärn, Mart Kals, Shuang Luo, Shanmukha Sampath Padmanabhuni, Marianna Niemi, Javier Gracia-Tabuenca, Mika Helminen, Tiina Luukkaala, Iida Vähätalo, Jyrki Tammerluoto, Sarah Smith, Tom Southerington, Petri Lehto

Clinical characteristics

Clinical characteristics of familial and sporadic NAH and cases with toxic thyroid nodules

*Family A was nonconsanguineous kindred of Polish origin, with overt NAH across three generations (***Figure 1***). The age at presentation of clinical symptoms and diagnosis ranged from 10 to 40 years. All patients had plasma TSH and free T4 (fT4) concentrations below and above the reference range, respectively, and undetectable levels of TSHR antibodies at diagnosis. The index case (#270) and his cousin (#271) were diagnosed at 13 and 10 years of age, respectively, and are currently being treated with a standard dose of methimazole. Three other family members (#268, #269 and #273) showed persistent hyperthyroidism, and underwent thyroidectomy and radioiodine treatments. Individual #270 presented acceleration of linear growth together with advanced bone age and a decrease in body weight at the time of diagnosis, typical for pediatric hyperthyroidism. There was no history of osteoporosis or fractures among the family members. Moreover, all other biochemical tests were normal in hyperthyroid cases* **(Figure 1 and Supplemental Table S1***).*

*Families B and C were Finnish nonconsanguineous kindreds. Every child had normal umbilical TSH values at neonatal screening for congenital hypothyroidism (CH) (***Supplemental Table S1***). All affected individuals were initially diagnosed with subclinical hyperthyroidism and had suppressed serum TSH, but normal fT4 and free T3 (fT3) levels. However, individuals #266, #279 and #280 developed typical hyperthyroid symptoms during several years of follow-up and a low/normal dose of antithyroid medication was initiated. All affected individuals had a relapse of hyperthyroidism after cessation of 12-18 months of antithyroid treatment. Antithyroid medication was continued in individual #279 and thyroidectomy was performed for individuals #280 and #266 at the ages of 46 and 19, respectively. Interestingly, two individuals (#266 and #279) of family B (***Supplemental Figure S1***) had mostly subclinical hyperthyroidism prior to pregnancy, which rapidly worsened during the first trimester of pregnancy with an increase in free T4 and T3 levels and required treatment adjustment. The radioiodine uptake of patient #266 was increased to 51% (reference range: 17 - 42%). The thyroid histology of patients #266 and #280 revealed typical thyroid hyperplasia with variable-sized follicles (***Supplemental Figure S2***).*

In the family C, the index patient (#74) underwent thyroid function tests due to essential hypertension at the age of 24 years. At diagnosis of subclinical hyperthyroidism, her TSH concentration was below detection limit (0.06 mU/L, reference: 0.5 - 3.6 mU/L), but fT4 (17 pmol/L, reference: 9.0 -19 pmol/l) and fT3 (5.5 pmol/L, reference: 2.6 - 6 pmol/L)

*concentrations were in range (***Figure 1***). The patient had no hyperthyroid symptoms, and thyroid imaging, bone density, or fT4 levels remained within the normal over 20 years of follow-up. The patient's son's (#75) serum TSH and fT4 levels were measured routinely, and subclinical hyperthyroidism was diagnosed based on suppressed serum TSH but normal fT4 concentrations. Follow-up revealed normal bone age, linear growth, BMI, head growth, weight gain, and neurological development (***Supplemental Figure S3 and Table S1***). Antibody tests for TSHR, thyroglobulin, and thyroid peroxidase were also negative, and thyroid ultrasound imaging was normal for both subjects in family C.*

*In the family D, the index case (#293) and his brother developed overt symptomatic NAH at the age of 13 months and 3 years, respectively, while the patients' mother and the sibling were asymptomatic. At the time of recruitment, his father had low serum TSH, but normal fT4 concentration with no hyperthyroid symptoms (***Figure 1***).*

Patients with sporadic nonautoimmune congenital hyperthyroidism and toxic thyroid nodules

*Patient #121 was born at 37 + 6 weeks of gestation as a third child to euthyroid parents with no history of thyroid disease (***Supplemental Table S1***). There were no complications or symptoms of hyperthyroidism during pregnancy or delivery, and no TSHR-stimulating antibodies were detected in the serum of the child or the mother. The umbilical cord blood TSH concentration, measured at birth during routine hypothyroidism screening, was below the detection limit. Diagnosis of hyperthyroidism was confirmed at the first day of life with very high serum fT4 concentration (73.3 pmol/L, reference: 10 - 36 pmol/l) and TSH below the detection limit (<0.03 mU/L) (***Figure 2A***). He had normal linear growth (***Figure 2B***), but head circumference was initially -2 SD in Finnish children but increased to + 2 SD (***Supplemental Figure S3***). A total thyroidectomy was performed at the age of 6.6 years due to the permanent requirement for antithyroid medication and high variation in thyroid function test, as illustrated in* **Figure 2A**. *Thyroidectomy led to severe complications including transient bilateral palsy of the laryngeal nerve, permanent hypocalcemia and significant weight gain (***Figure 2C***). While thyroid ultrasound showed a normal size and location, the iodine uptake test revealed a relatively high accumulation of radioactivity in both lobes (***Figure 2D***). Histological analysis of the thyroid showed normal follicles of variable size, several follicles with vacuoles, and homogenous colloid staining (***Figure 2E***).*

We studied two children (#229 and 230) with typical hyperthyroid symptoms, no stimulating TSHR antibodies and overt hyperthyroidism in thyroid function tests at diagnosis

(serum TSH levels 0.06 and <0.03 mU/L, reference: 0.51 - 4.3 mU/L; fT4 23.6 and 31.5 pmol/L, reference: 7.7 -12.6 pmol/L) at the age of 12 and 10 years, respectively. Thyroid ultrasound revealed a single nodule in the right lobe (#229, **Figure 3A***) and multiple nodules and hemigoiter in the other patient (#230). In both cases, the thyroid* ^{99m}*Tc pertechnetate scan revealed high radioactive tracer uptake specifically in the nodules (***Figure 3B***). The patients underwent hemithyroidectomy without complications, and hyperthyroidism was resolved. Histological analysis revealed that the nodules consisted of follicles of variable sizes, with only a small amount of colloid, a thick thyrocyte epithelial layer (***Figure 3C***), and diffuse hyperplastic thyrocytes in multiple nodules (***Figure 3D***).*

Genetic screening of *TSHR* mutations in patients with CH or resistance to TSH

We previously utilized a thyroid gene panel to screen for mutations in individuals with congenital hypothyroidism (CH) (15) and found twins with a heterozygous variant in TSHR (Arg519Cys) (#67 and 68, **Supplemental Table S2***). Here, we performed segregation analysis of the family members and found that the father (#137) was a euthyroid carrier and lacked an additional rare thyroglobulin variant (TG, c.6952G>A, p.Asp2318Asn), that the twins and euthyroid mother possessed (#139,* **Table S2***). Furthermore, in a new cohort of 30 patients with CH or resistance to TSH (RTSH), we identified a known pathogenic homozygous TSHR mutation (p.Pro162Ala) (16) in a child with RTSH diagnosed before 7 years of age (#143,* **Supplemental Tables S1 and S2***). The patient's parents were heterozygous carriers with slightly elevated serum TSH concentrations (#144 and #145,* **Supplemental Table S1***). Moreover, the same heterozygous TSHR p.Pro162Ala mutation was detected in a 3-year-old patient with RTSH (TSH: 10-12 mIU/L, reference: 0.5 - 3.6 mIU/L, and normal fT4), with negative TPO autoantibody tests and normal neonatal TSH screening.*

Extended structural and mechanistic insights into the I640V and S273N CAMs

To explore the mechanism leading to constitutive receptor activation by the newly identified natural variant p.I640V, some general and detailed aspects of receptor activation, previously (17) and recently (12) received, must be considered. Activation of the TSHR and other GPHRs is related to hormone binding (or activating antibodies in the case of TSHR), which induces a strong relative movement of specific extracellular parts (red arrows in **Figure S8C,D)***, constituted by the LRRD and the hinge region (***Figure S8A versus S8B***). This "ridged body movement" leads to strong modifications in the disulfide bridge linked more flexible hinge part directly adjacent to TMH1. This C-terminal hinge part is the p10 region with highly essential amino acids for receptor activity regulation. The p10 is a subunit of a so-called "intramolecular agonistic unit" comprised of diverse receptor fragments from the LRRD (e.g. Ser281), the hinge region (Asp403-Gly414), and the TMD (e.g. EL2, TMH7) assembled in a tight spatial unit. Comparison of molecular details between the new inactive and active state TSHR structures reveal, that amino acid residues of the p10 region (e.g. D403 to N406) are released from interactions involved in maintaining the inactive state (termed here "inactivator component") (***Figure S8C***, not shown in details), which is supported by CAMs reported at these positions (18). In consequence, Phe405 centrally located between EL2 and TMH7, gets dislocated and shifts upwards, whereby a tight hydrophobic package of amino acids surrounding this phenylalanine gets interrupted (e.g. Ile568, Met572). This process is accompanied or enables a shift in the C-terminal part of the structurally flexible p10 region (Glu409) towards TMH7 (Lys660), which moves inward to the central core of the TMD. This re-arrangement is then stabilized by a newly formed Glu409-Lys660 salt bridge (between hinge (termed here "activator component") -TM7) (***Figure S8D***). This extracellular process, which is in principal agreement to the previously supposed switching of the ectodomain from a tethered inverse agonist to an agonist, can now be determined more precisely to a spatially very restricted "intramolecular ligand". Modulation of this finally leads to strong modifications in the close TMH7-TMH6 interface, which is essential for maintaining the receptor in an inactive and in the active state conformations. The relative movement of TMH7 during p10 activation causes a loss of interactions one-turn below Lys660 (membrane-embedded) between Leu633, Tyr667 (TMH7), and Ile640 in TMH6. Thereby induced movement of the Ile640 side chain is directly coupled to Pro639 (according to the unifying Ballesteros & Weinstein class A GPCR numbering system P6.50), which participates in the constitution of the TMH6 helix-kink that is pivotal for TMH6 outward movement during receptor activation. This TMH6 movement – a hallmark of class A GPCR activation in G-protein coupling - is*

further reflected and supported by a strong dislocation of the Met637 side chain, which is in the group of GPHRs at position 6.48 instead of the more class A GPCR common Trp/Phe6.48 (toggle switch tryptophan).

The mutation p.S237N is specifically interesting as it is located at the extracellular leucine rich repeat (LRR) 9, whereby the entire LRR domain is constituted by 11 repeats (amino acids Cys24-Asn288, **Figure S8C***). Moreover, Ser237 is not in the hormone binding site and not in a direct contact to any other side chain in the LRRD or in the TMD. This is in stark contrast to the known extracellular CAMs in position Ser281 (48–50). Ser281 is close to the TMD and especially the EL1, which brings the Ser281 side chain in direct spatial relation to the tethered ligand-associated activation mechanism. This explains from a structural perspective why at this residue receptor activation (red arrows in* **Figure S8C, D** *indicating structural shifts from inactive to active state conformations) can be initiated by a mutation in contrast to position Ser237 without any intra- or intermolecular interaction partner. Of note, further side-chain variations p.S237A, p.S237D, p.S237V did not result in constitutive activation, and this TSHR CAM cannot be hyperstimulated by an increased affinity of CG to TSHR (***Figure 3D***), which altogether supports a specific activation mechanism for variant p.S237N.*

References:

1. Tanner JM, Gibbons RD. A Computerized Image Analysis System for Estimating Tanner-Whitehouse 2 Bone Age. Horm Res Paediatr. 1994;42(6):282–287.
2. Tsunekawa K, et al. Identification and Functional Analysis of Novel Inactivating Thyrotropin Receptor Mutations in Patients with Thyrotropin Resistance. 2006.
3. Führer D, et al. Identification of a new thyrotropin receptor germline mutation (Leu629Phe) in a family with neonatal onset of autosomal dominant nonautoimmune hyperthyroidism. Journal of Clinical Endocrinology and Metabolism. 1997;82(12):4234–4238.
4. Grasberger H, et al. Brief report: A familial thyrotropin (TSH) receptor mutation provides in vivo evidence that the inositol phosphates/Ca2+ cascade mediates TSH action on thyroid hormone synthesis. Journal of Clinical Endocrinology and Metabolism. 2007;92(7):2816–2820.
5. Russo D, et al. Detection of an Activating Mutation of the Thyrotropin Receptor in a Case of an Autonomously Hyperfunctioning Thyroid Insular Carcinoma1. J Clin Endocrinol Metab. 1997;82(3):735–738.
6. Arseven OK, et al. Substitutions of tyrosine 601 in the human thyrotropin receptor result in increase or loss of basal activation of the cyclic adenosine monophosphate pathway and disrupt coupling to Gq/11. Thyroid. 2000;10(1):3–10.
7. Torban E, Pelletier J, Goodyer P. F329L polymorphism in the human PAX8 gene. Am J Med Genet. 1997;72(2):186–187.
8. Akcurin S, et al. A family with a novel TSH receptor activating germline mutation (p.Ala485Val). Eur J Pediatr. 2008;167(11):1231–1237.
9. Bodian DL, et al. Germline Variation in Cancer-Susceptibility Genes in a Healthy, Ancestrally Diverse Cohort: Implications for Individual Genome Sequencing. PLoS One. 2014;9(4):94554.
10. Korevaar TIM, et al. ENDOCRINE EPIDEMIOLOGY Reference ranges and determinants of total hCG levels during pregnancy: the Generation R Study. Eur J Epidemiol. 2015;30:1057–1066.
11. Robinson JT, et al. Integrative genomics viewer. Nat Biotechnol. 2011;29(1):24–26.
12. Faust B, et al. Autoantibody mimicry of hormone action at the thyrotropin receptor. Nature. 2022;609(7928):846–853.
13. Duan J, et al. Hormone- and antibody-mediated activation of the thyrotropin receptor. Nature. 2022;609(7928):854–859.
14. Jaeschke H, et al. Hyperthyroidism and Papillary Thyroid Carcinoma in Thyrotropin Receptor D633H Mutant Mice. Thyroid. 2018;28(10):1372–1386.
15. Löf C, et al. Detection of Novel Gene Variants Associated with Congenital Hypothyroidism in a Finnish Patient Cohort. Thyroid. 2016;26(9):1215–1224.
16. Sunthornthepvarakul T, et al. Thyrotropin Caused By Mutations in. N Engl J Med. 1995;332(3):155–160.
17. Kleinau G, et al. Structural-functional features of the thyrotropin receptor: A class A G-protein-coupled receptor at work [p. Front Endocrinol (Lausanne). 2017;8(APR). https://doi.org/10.3389/fendo.2017.00086.
18. Mueller S, et al. Significance of ectodomain cysteine boxes 2 and 3 for the activation mechanism of the thyroid-stimulating hormone receptor. Journal of Biological Chemistry. 2006;281(42):31638–31646.
